# Integrated multi-omics analyses reveals molecules governing sperm metabolism potentially influence bull fertility

**DOI:** 10.1038/s41598-022-14589-w

**Published:** 2022-06-23

**Authors:** Thirumala Rao Talluri, Arumugam Kumaresan, Manish Kumar Sinha, Nilendu Paul, John Peter Ebenezer Samuel King, Tirtha K. Datta

**Affiliations:** 1 Theriogenology Laboratory, Veterinary Gynaecology and Obstetrics, Southern Regional Station of ICAR- National Dairy Research Institute, Bengaluru, Karnataka 560030 India; 2 Animal Genomics Laboratory, ICAR - National Dairy Research Institute, Karnal, Haryana 132 001 India

**Keywords:** Biotechnology, Molecular biology, Systems biology

## Abstract

Bull fertility is of paramount importance in bovine industry because semen from a single bull is used to breed several thousands of cows; however, so far, no reliable test is available for bull fertility prediction. In the present study, spermatozoa from high- and low-fertility bulls were subjected to high-throughput transcriptomic, proteomic and metabolomic analysis. Using an integrated multi-omics approach the molecular differences between high- and low-fertility bulls were identified. We identified a total of 18,068 transcripts, 5041 proteins and 3704 metabolites in bull spermatozoa, of which the expression of 4766 transcripts, 785 proteins and 33 metabolites were dysregulated between high- and low-fertility bulls. At transcript level, several genes involved in oxidative phosphorylation pathway were found to be downregulated, while at protein level genes involved in metabolic pathways were significantly downregulated in low-fertility bulls. We found that metabolites involved in Taurine and hypotaurine metabolism were significantly downregulated in low-fertility bulls. Integrated multi-omics analysis revealed the interaction of dysregulated transcripts, proteins and metabolites in major metabolic pathways, including Butanoate metabolism, Glycolysis and gluconeogenesis, Methionine and cysteine metabolism, Phosphatidyl inositol phosphate, pyrimidine metabolism and saturated fatty acid beta oxidation. These findings collectively indicate that molecules governing sperm metabolism potentially influence bull fertility.

## Introduction

Bull fertility is the most important economic trait in cattle because semen from one bull is used to artificially breed a large number of cows^1^. Use of semen from low-fertility bulls leads to colossal loss to the farmers; therefore selection of bulls for high fertility is very crucial for successful dairying. Currently, bulls are selected based on breeding soundness evaluation (BSE) that mainly focus on examination of phenotypic characteristics and evaluation of few characteristics of spermatozoa like motility and morphology. The bulls that passed all these criteria of breeding soundness evaluation still showed differences in conception rate to the magnitude of 20–25% among themselves^2–4^. Over the past decades, in vitro tests to screen the structural and functional integrity of the spermatozoon has increased markedly, but the capacity to estimate the fertility of a semen sample or of the sire from which it has been collected has not^5^. The methods for assessment of sperm phenotypic and functional characteristics have limited to moderate correlations with fertility and therefore are only of limited value for prediction of bull fertility^6^. Further, it has been shown that bulls with normal spermiogram, kinematics and morphology varied in their fertility^7^; the fertility of a considerable proportion of bulls remained suboptimal with a conception rate ranging from 20 to 45%^8^ leading to considerable economic losses. On the other hand, female fertility has received much attention in the last few decades, while bull fertility has been largely overlooked^9^.

Routinely, the bull fertility is estimated based on insemination of large number of cows and assessment of non-return rates/conception rates, which is costly and highly time consuming. Therefore, development of alternate techniques/methods/tools for bull fertility prediction is an area of research that has been active over a period of recent times. Recent developments in high-throughput sequencing technologies that allow the assessment of thousands of molecules in a given time has revolutionized the research on bull fertility. Using such techniques, several studies identified that bull fertility was influenced by several factors including sperm functional attributes^3, 10^, sperm DNA integrity^11, 12^, sperm transcripts^13–16^, sperm proteins^1, 2, 17, 18^, seminal plasma proteins^19–21^ and sperm metabolites^20, 22, 23^. The major advancements in the -*omic* technologies (metabolomics, proteomics, transcriptomics, and genomics) have enabled high-throughput screening of a wide range of molecular and cellular dynamics in fertility molecules^24^. Employing these techniques several studies identified the molecular differences between the spermatozoa of high- and low fertility bulls^2, 14, 16, 22, 23^. However, it is pertinent to mention here that the most of the earlier studies conducted to identify bull fertility markers have employed assessment of a particular parameter (for instance, either sperm transcripts or proteins) in relation to bull fertility. Although accumulating studies indicate that the level of specific sperm molecules can be related to bull fertility; so far no single, highly reliable test is available^25^.

Interpreting the biological results from data of a single type of -*omics* is challenging due to the complex biochemical regulation involved at multiple levels. Integration of multi-omics data offers several advantages; the biological processes can be investigated in a more comprehensive manner at the transcript and protein levels that regulate the underlying pathway mechanisms. Further, it has the potential to reveal key biological insights into pathways that would otherwise not be made apparent through single-omics studies. Therefore, to develop highly sensitive biomarkers or diagnostic tools for the evaluation of male fertility, an array of high throughput technologies emerging from *multi-omics* or integrative approaches has to be employed^26, 27^. Therefore, in the present study, we adopted an integrated multi-omics analysis approach combining transcripts, proteins, and metabolites data from high- and low-fertility bull spermatozoa, for understanding the critical sperm molecular differences associated with bull fertility. To the best of our knowledge this is first study to use integrated multi-omics approach to understand the bull fertility. We report here the differential expression levels of sperm RNA, proteins, and metabolites between high- and low-fertility breeding bulls, and the biological insights into key pathways associated with bull fertility.

## Results

### Seminal parameters in high- and low-fertility bulls

The proportion of membrane intact spermatozoa was lower (P < 0.05) in LF bulls in comparison to the HF bulls. Similarly, the proportion of acrosome intact spermatozoa was also significantly (P < 0.01) higher in HF bulls compared to LF bulls. While the proportion of spermatozoa with high mitochondrial membrane potential was higher (P < 0.05) in HF bulls, there was no difference observed in the proportion of spermatozoa with high intracellular calcium between the two groups of bulls (Supplementary Fig. 1).

### Sperm transcriptomic profile

**Figure 1:**
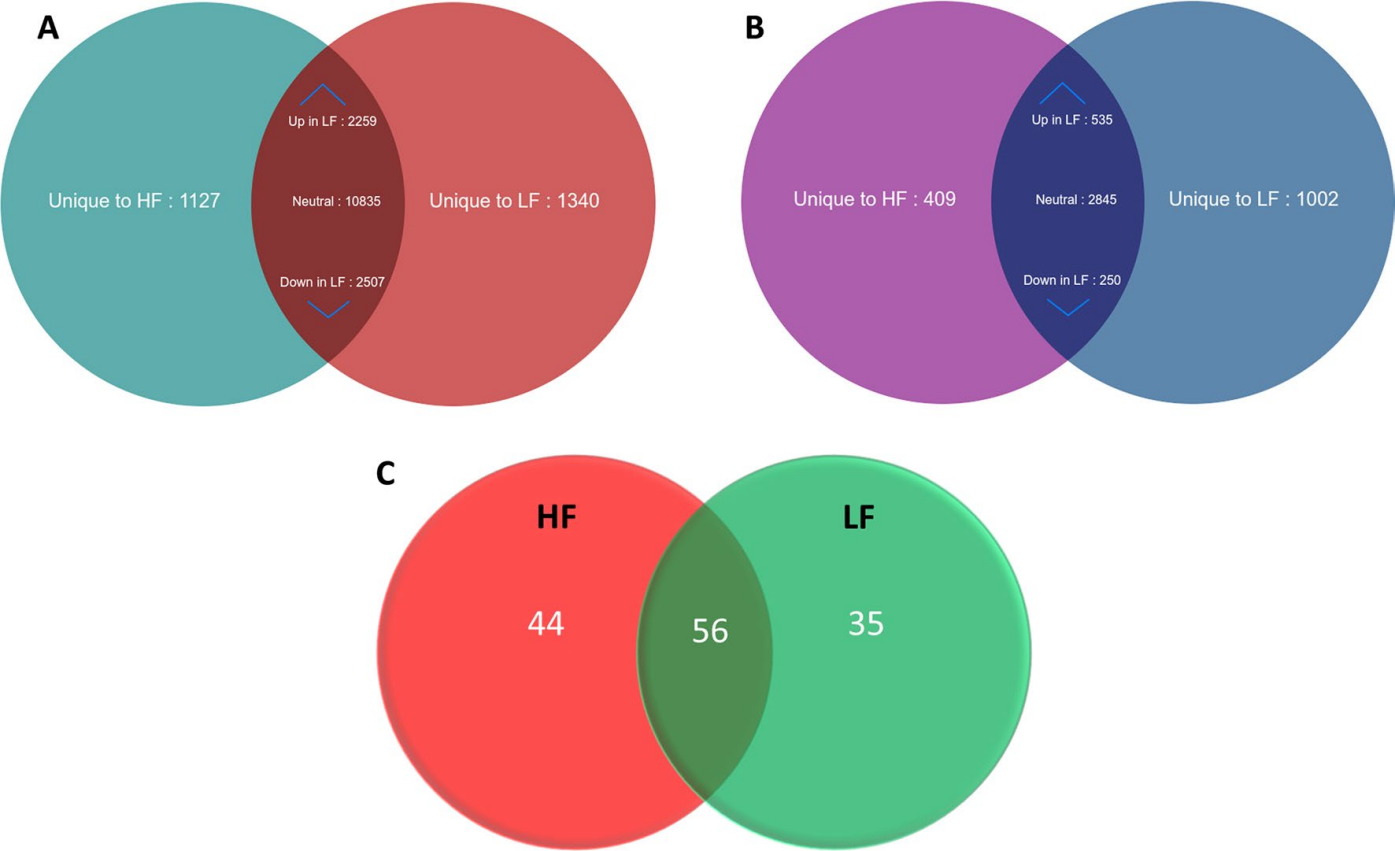
Venn diagram representing total number of transcripts (**A**), proteins (**B**) and metabolites (**C**) detected in high- and low-fertility bull spermatozoa. *HF* high-fertility, *LF* low-fertility bulls. The number of upregulated (Up) and downregulated (Down) transcripts and proteins are also indicated in the figure.

The sequencing library resulted in generation of Paired-end (76 bp × 2) *fastq* file; on an average, 28.7 million reads were generated per sample. After processing the raw data using *Cutadapt* for quality, adapters and length filtering, an average of 24 million reads per sample was mapped against the *Bos taurus* genome. The read count obtained from *HTSeq*-count tool was taken as an input for *DESeq* tool for analysis of differential expression of the transcripts. A total number of 16,728 and 16,941 transcripts were detected in spermatozoa from high- and low-fertility bulls. A total number of 15,601 transcripts were common to both high- and low-fertility bull spermatozoa, while 1127 and 1340 transcripts were unique to high- and low-fertility bull spermatozoa (Fig. 1A). A total of 4766 transcripts were found to be dysregulated between the two groups among which, 383 transcripts (219 upregulated and 164 downregulated) were found highly dysregulated (P ≤ 0.05). The top 10 upregulated and downregulated transcripts in low-fertility bulls are listed in Table 1. For determining the distribution of transcripts between the both the groups, volcano plot was developed, which represented the difference (expressed in base-2 log ratio of transcript abundance − intensity) between the two samples compared (*x*-axis), and the significance level associated to this value (*y*-axis). The demarcation in expression levels of sperm transcripts belonging to high- and low-fertility bulls are shown as volcano plot in Supplementary Fig. 2A. The list of dysregulated transcripts observed in the current study, along with the p values, are provided in the Supplementary File 1.

**Table 1:** Top 10 upregulated and down regulated transcripts in low-fertility bulls.

S. no.	Transcript ID (Ensemble)	Gene	Gene description	Log2 (fold change)	Reported function
**Top 10 upregulated transcripts in low-fertility bulls**					
1.	ENSBTAT00000013402	TPT1	Translationally-controlled tumor protein (TCTP)	8.53	Calcium ion binding
2.	ENSBTAT00000006465	PFN1	Profilin-1 (Profilin I)	6.76	Actin cytoskeleton organization; positive regulation of actin filament bundle assembly
3.	ENSBTAT00000008132	ACTG1	Actin, cytoplasmic 2 (Gamma-actin) [Cleaved into: Actin, cytoplasmic 2, N-terminally processed]	6.30	ATP binding
4.	ENSBTAT00000018491	RPS11	40S ribosomal protein S11	6.14	rRNA binding; structural constituent of ribosome
5.	ENSBTAT00000021033	RPL18A	60S ribosomal protein L18a	5.99	Cytoplasmic translation
6.	ENSBTAT00000007429	TMSB10	Thymosin beta-10 (Thymosin beta-9) [Cleaved into: Thymosin beta-8]	5.93	Actin filament organization; regulation of cell migration
7.	ENSBTAT00000003962	RPS3	40S ribosomal protein S3 (EC 4.2.99.18)	5.80	Apoptotic process; DNA repair; positive regulation of apoptotic signaling pathway
8.	ENSBTAT00000004188	RPL3	60S ribosomal protein L3	5.75	RNA binding; structural constituent of ribosome
9.	ENSBTAT00000005581	EEF2	Elongation factor 2 (EF-2)	5.74	GTPase activity; GTP binding; ribosome binding;
10.	ENSBTAT00000017497	HSPA8 HSC70	Heat shock cognate 71 kDa protein (Heat shock 70 kDa protein 8)	5.22	ATPase activity; ATP binding; clathrin-uncoating ATPase activity
**Top 10 downregulated transcripts in low-fertility bulls**					
1.	ENSBTAT00000027868	UPF1	UPF1, RNA helicase and ATPase	− 5.62	ATP binding; chromatin binding; RNA binding; RNA helicase activity; telomeric DNA binding; zinc ion binding
2.	ENSBTAT00000008738	TSSK6	Testis specific serine kinase 6	− 5.62	ATP binding; magnesium ion binding; protein-containing complex binding; protein serine/threonine kinase activity
3.	ENSBTAT00000013608	ICAM1	Intercellular adhesion molecule 1	− 5.81	Cell–cell adhesion
4.	ENSBTAT00000002707	CDC34	Uncharacterized protein	− 5.92	ATP binding; ubiquitin conjugating enzyme activity
5.	ENSBTAT00000005688	MRPL21	39S ribosomal protein L21, mitochondrial	− 6.00	Mitochondrial activity
6.	ENSBTAT00000063495	FAM57A	Family with sequence similarity 57 member A (Hypothetical LOC508425)	− 6.39	Lipid homeostasis
7.	ENSBTAT00000026782	AQP7	Uncharacterized protein	− 6.53	Urea transmembrane transporter activity
8.	ENSBTAT00000044628	ANKRD9	ANKRD9 protein	− 6.85	Protein ubiquitination
9.	ENSBTAT00000049255	TEX264	Testis expressed 264	− 6.89	Autophagosomal membrane closure
10.	ENSBTAT00000012504	LUZP1	Uncharacterized protein	− 8.33	Uncharacterized protein

### GO, KEGG and network analysis

**Figure 2:**
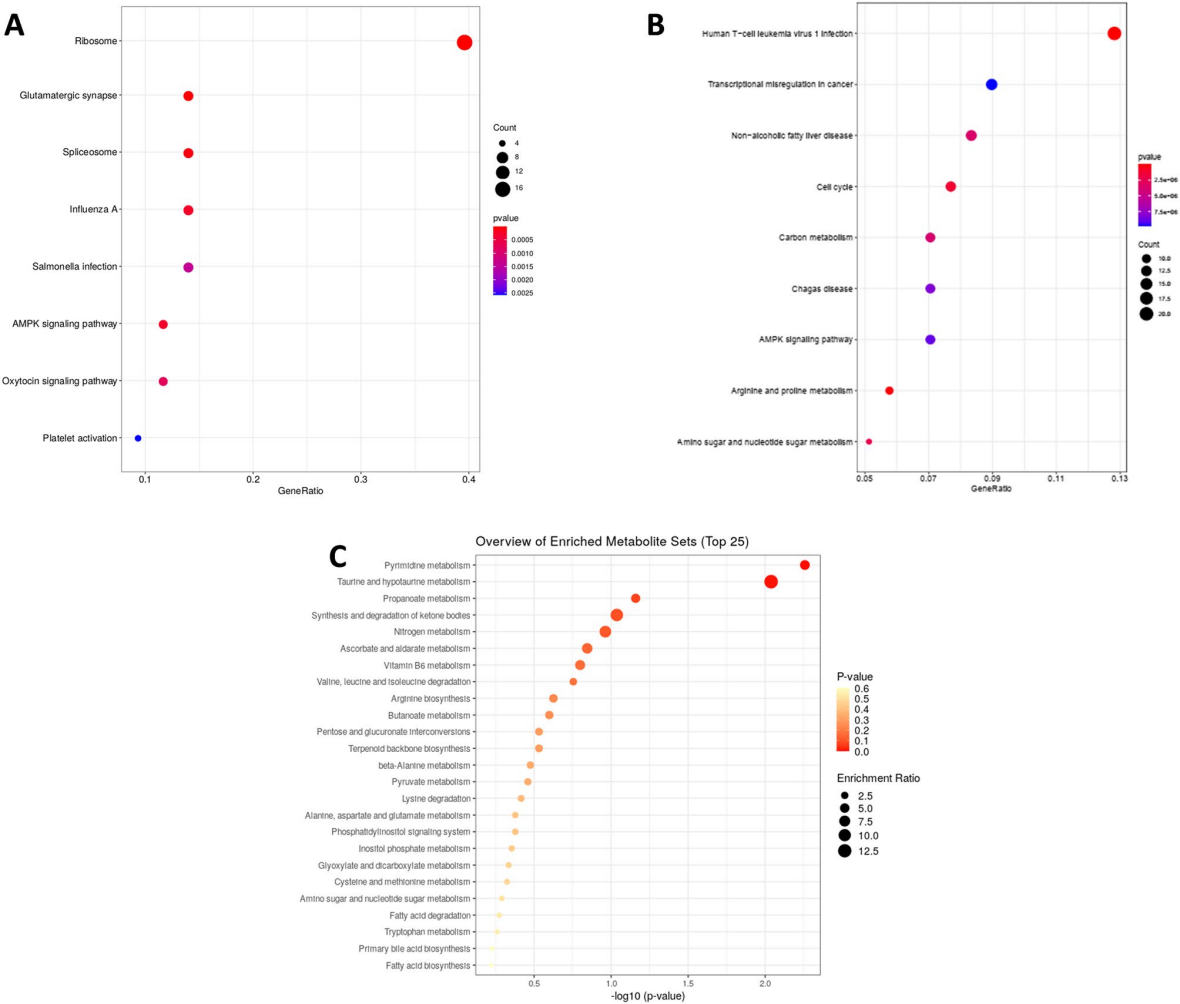
KEGG (Kyoto Encyclopedia of Genes and Genomes) pathway enrichment analysis of dysregulated transcripts (**A**), proteins (**B**) and metabolites (**C**) between high- and low-fertility bulls.

We investigated the potential biological functions of the differentially expressed genes (DEGs) (P ≤ 0.05). Gene ontology (GO) analysis of total 383 DEGs revealed their involvement in 25 BPs, 14 MFs, and 27 CCs (the top 10 in each GO category are shown in Supplementary Fig. 3A and 6 KEGG pathways (Fig. 2A). Majority of the transcripts are localised in nucleus, extracellular exosome, ribosomal unit, nucleolus and mitochondrial inner membrane. KEGG pathway enrichment analysis revealed that the differentially expressed transcripts were involved in pathways related to ribosome pathway (4.89%, 17 counts, 2.34E−09), AMPK Signalling pathway (2.01%, 7 counts), Glutamatergic synapse (1.72%, 6 counts) and Spliceosome (1.72%, 6 counts). The GO analysis showed that the DEGs were enriched in molecular functions like Ribosomal activity, RNA binding, ATP binding and actin binding (Supplementary Fig. 3A). Network analysis of total dysregulated sperm transcripts revealed their involvement in 24 BPs (Supplementary Fig. 4A) and important pathways like negative regulation of cell motility, tube formation, protein folding, hormone mediated signalling pathway and histone binding etc.

### Sperm proteomic profile

LC–MS/MS analysis detected a total of 5041 proteins in bull spermatozoa. A total of 4039 and 4632 proteins were found in HF and LF bull spermatozoa, respectively and 3630 proteins were found common in both the groups. A total of 409 were unique to HF and 1002 proteins were unique to LF bull spermatozoa (Fig. 1B) based on the normalized spectral abundance factor (NSAF) ratio and protein abundance. A total of 785 proteins were found to be differentially expressed proteins (DEPs) between high- and low-fertility bull spermatozoa. The list of top 10 up regulated and top 10 down regulated proteins in low fertility group are given in Table 2. The list of dysregulated proteins observed in the current study and with their fold change are provided in the Supplementary File 2.

**Table 2:** Top 10 upregulated and down regulated proteins in low-fertility bulls.

Sr. no.	Uniprot ID	Gene	Gene description	Log2 (fold change)	Reported function
**Top ten upregulated proteins**					
1.	Q3T168	CACYBP	Calcyclin-binding protein	2.38	Ubiquitin protein ligase binding
2.	C0LZJ1	DMRT1	Doublesex and mab-3 related transcription factor 1	2.4	Germ cell development; male germ cell proliferation; male sex determination; male sex differentiation, Sertoli cell differentiation; sex differentiation
3.	P80227	APEH	Acylamino-acid-releasing enzyme	2.42	Serine-type endopeptidase activity
4.	Q2KHT6	FBXO32	F-box only protein 32	2.53	Protein ubiquitination
5.	O19131	TNFRSF1A	Tumor necrosis factor receptor superfamily member 1A	2.55	Apoptotic process; cell surface receptor signaling pathway; cytokine-mediated signaling pathway; defense response; prostaglandin metabolic process
6.	P21214	TGFB2	Transforming growth factor beta-2 proprotein	2.59	Activation of protein kinase activity; BMP signaling pathway; negative regulation of alkaline phosphatase activity; negative regulation of cell growth; type III transforming growth factor beta receptor binding; type II transforming growth factor beta receptor binding
7.	Q0VCA8	HAAO	3-hydroxyanthranilate 3,4-dioxygenase	2.7	Ferrous iron binding
8.	A5PJZ1	SLC25A24	Calcium-binding mitochondrial carrier protein SCaMC-1	2.84	Cellular response to calcium ion; cellular response to oxidative stress; mitochondrial transport; regulation of cell death
9.	Q3T0I4	ALYREF	THO complex subunit 4	3.21	mRNA processing; mRNA transport; RNA export from nucleus; RNA splicing
10.	Q3ZC33	GULO	l-gulonolactone oxidase	5.8	l-ascorbic acid biosynthetic process
**Top ten downregulated proteins**					
1.	Q3T144	TMEM106C	Transmembrane protein 106C	− 3.93	Integral component of Endoplasmic reticulum, facilitates sperm motility
2.	O18883	TXNDC9	Thioredoxin domain-containing protein 9	− 3.35	Microtubule organization, protection against oxidative stress
3.	Q08DX7	SPNS1	Protein spinster homolog 1	− 3.17	Transmembrane transporter activity
4.	Q3SZ84	BOLA3	BolA-like protein 3	− 3.07	Mitochondrial function
5.	P61284	RPL12	60S ribosomal protein L12	− 2.9	Structural constituent of ribosome
6.	Q2NL16	FBXO28	F-box only protein 28	− 2.88	Protein polyubiquitination
7.	O97552	POU5F1	POU domain, class 5, transcription factor 1	− 2.55	DNA binding; DNA-binding transcription factor activity, RNA polymerase II-specific, Blastocyst formation
8.	Q5BIS9	PRKAB1	5ʹ-AMP-activated protein kinase subunit beta-1	− 2.51	Protein kinase activity; protein kinase binding
9.	Q5EA49	MIS12	Protein MIS12 homolog	− 2.49	Attachment of mitotic spindle microtubules to kinetochore; cell division;
10.	Q9TS87	TAGLN	Transgelin	− 2.34	Actin filament binding

### GO, KEGG and network analysis of proteome

The biological roles of the DEPs were analysed through GO and KEGG analysis. It was found that the DEPs are involved in 80 BPs, 36 CCs, 33 MPs and 24 KEGG pathways. The principle KEGG pathways of DEPs were found to be enriched in various metabolic pathways, cell cycle, carbon metabolism, AMPK Signalling metabolism, cGMP-PKG signalling pathway, Glycolysis/Gluconeogenesis metabolism and arginine proline metabolism (Fig. 2B). GO analysis revealed the role of DEPs in various biological process like oxidation reduction, cell differentiation, proteolysis, positive regulation of apoptosis and phosphorylation (Supplementary Fig. 3B). The identified metabolic functions for the DEPs were actin binding, calmodulin binding, Protein kinase binding and histone acetylase binding, and the major cellular components were associated with cytoplasm, nucleus and mitochondria inner and outer membranes. Proteins with a significant quantitative change (P < 0.05) were considered as significantly differentially expressed between the two groups. The intensity values of both the groups were considered for the volcano plot, in which there was a clear difference in the expressions of proteins between groups (Supplementary Fig. 2B). Upon network bioinformatics analysis, it was identified that involvement of maximum DEPs in sperm motility, single fertilisation, Glycolysis/gluconeogenesis and execution of phase of apoptosis (Supplementary Fig. 4B).

### Sperm metabolomic profile

Metabolomic profiling using mass spectroscopy revealed a total of 3704 metabolites in bull spermatozoa. After excluding the exogenous metabolites, 44 metabolites were found to be unique to HF and 35 to be unique in LF group (Fig. 1C). A total of 56 metabolites were observed to be common to both the groups and among these 33 metabolites were detected to be dysregulated (DEM). Top 10 upregulated and down regulated metabolites among the total dysregulated metabolites are listed in Table 3. Dysregulated metabolites (P ≤ 0.05) (33) were subjected for Metabolite Set Enrichment Analysis to identify their role in important pathways (Fig. 2C). The potential pathways identified were pyrimidine metabolism, Taurine and hypotaurine metabolism, propanoate metabolism, pyruvate metabolism, synthesis and degradation of ketone bodies and nitrogen metabolism. The list of dysregulated metabolites (P ≤ 0.05) and their fold change are provided in the Supplementary File 3.

**Table 3:** Top 10 upregulated and down regulated metabolites in low-fertility bulls.

S. no.	ID	Metabolite	Log2 (fold change)	Metabolism
**Top 10 upregulated metabolites**				
1.	HMDB0000965	Hypotaurine	6.2286	Taurine and hypotaurine metabolism
2.	HMDB0004122	Selenocystine	3.5978	Selenium metabolism
3.	HMDB0003537	DITP	3.2176	Purine metabolism
4.	HMDB0000156	l-malic acid	3.1025	Citrate cycle (TCA cycle), Pyruvate metabolism, Glyoxylate and dicarboxylate metabolism
5.	HMDB0001191	Deoxyuridine triphosphate	2.4029	Pyrimidine metabolism
6.	HMDB0001096	Carbamoyl phosphate	2.2636	Nitrogen metabolism, Arginine biosynthesis, Alanine, aspartate and glutamate metabolism
7.	HMDB0012238	Iodide	2.0618	Thyroxine metabolism
8.	HMDB0001011	Methacrylyl-CoA	2.0549	Valine, leucine and isoleucine degradation
9.	HMDB0033458	N-carbamoylputrescine	1.9252	Arginine and proline metabolism
10.	HMDB0003369	CDP-alpha-d-Glucose	1.6811	Starch and sucrose metabolism, amino sugar and nucleotide sugar metabolism
**Top 10 down regulated metabolites**				
1.	HMDB0001021	Malyl-CoA	− 0.98359	Glyoxylate and dicarboxylate metabolism, Methane metabolism
2.	HMDB0011104	3-Phosphoadenylylselenate	− 1.0946	Selenoamino acid metabolism
3.	HMDB0034259	3-Nitropropanoic acid	− 1.1658	Beta-Alanine metabolism
4.	HMDB0001142	FMNH2	− 1.5893	Riboflavin metabolism, Biosynthesis of cofactors
5.	HMDB0001484	Acetoacetyl-CoA	− 1.811	Synthesis and degradation of ketone bodies Butanoate metabolism, Terpenoid backbone biosynthesis, Pyruvate metabolism, Propanoate metabolism
6.	HMDB0003419	Formyl-CoA	− 2.0431	Glyoxylate and dicarboxylate metabolism
7.	HMDB0000652	Chondroitin 4-sulfate	− 2.3791	Glycosaminoglycan degradation
8.	HMDB0002346	Lactyl-CoA	− 3.3451	Propanoate metabolism
9.	HMDB0002127	3-Mercaptolactic acid	− 3.479	Cysteine and methionine metabolism
10.	HMDB0003417	d-cysteine	− 4.8072	Cysteine and methionine metabolism

### Integrated analysis of sperm transcriptome, proteome and metabolome in relation to bull fertility

**Figure 3:**
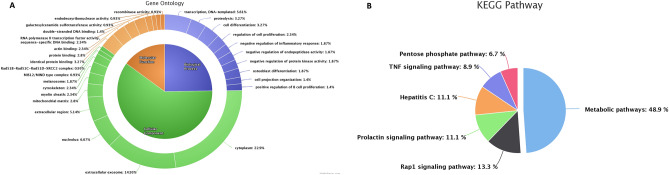
Gene Ontology and KEGG pathway analysis of translated proteins (overlap between transcripts and proteins). (**A**) Top 10 gene ontology categories. (**B**) KEGG pathway enrichment analysis.

**Figure 4:**
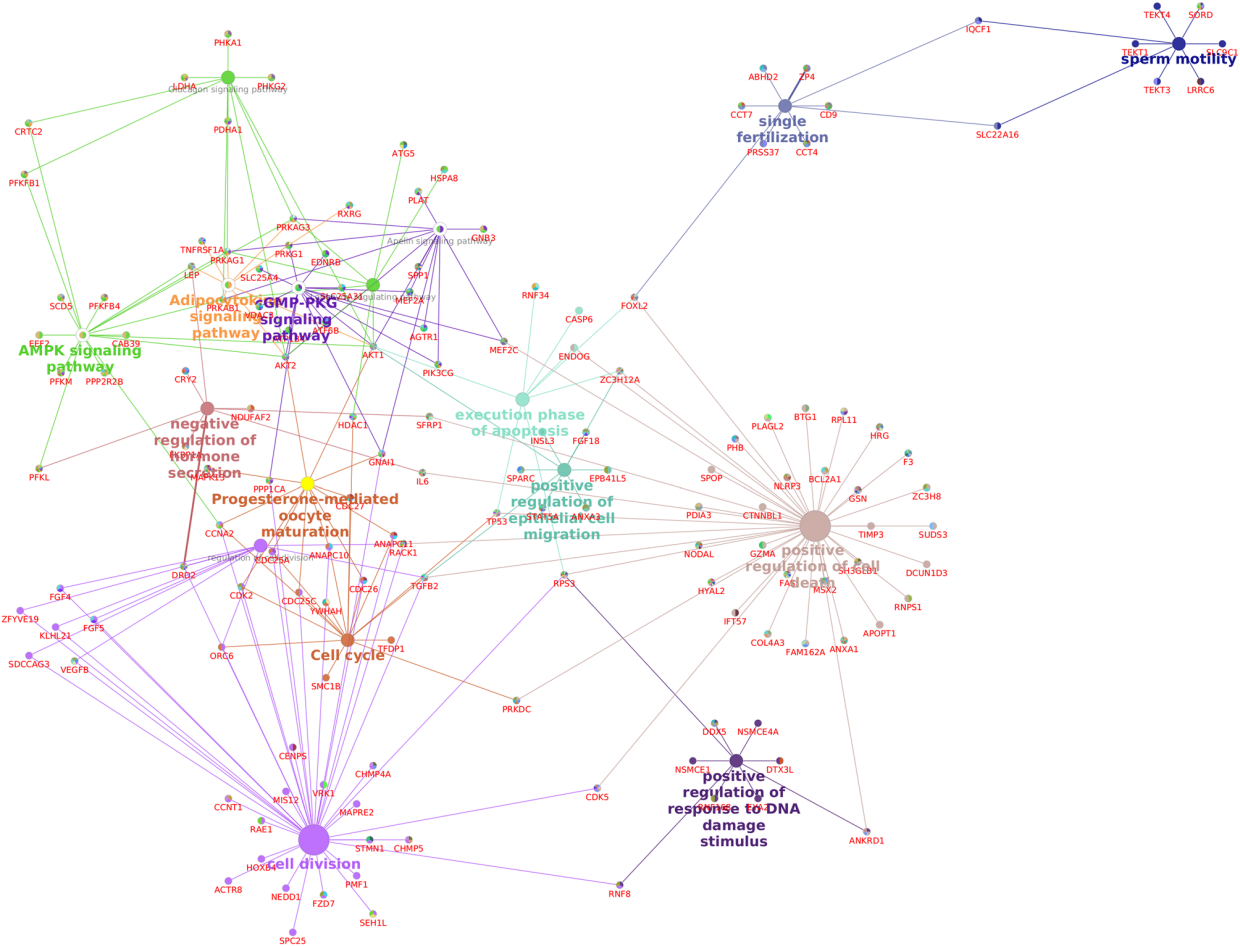
Interaction network of dysregulated transcripts and proteins.

The total transcripts obtained through NGS and total proteins detected through LC–MS/MS were compared to study the proportion of transcripts that are translated in the spermatozoa. A total of 4114 proteins were found to have corresponding transcripts and a total of 927 proteins were found to be unique. Subsequently, we identified that 182 dysregulated proteins had the corresponding transcripts, whose expression were also dysregulated between high- and low-fertility bulls. The functional annotation of these translated proteins revealed their association in 19 BP, 8 MF, 11 CC and involved in 6 KEGG pathways (P ≤ 0.05). The GO analysis of these translated proteins showed that these proteins were enriched in BPs, mainly including transcription, proteolysis, cell differentiation and negative regulation of protein kinase activity (Fig. 3A). The KEGG pathway enrichment analysis revealed that the translated proteins were mainly involved in pathways related metabolism, Rap1 signalling pathway, Prolactin signalling pathway, TNF signalling pathway and Pentose phosphate pathway (Fig. 3B). The integrated transcripts and proteome network is depicted in Fig. 4. The network analysis of transcriptome and proteome revealed that the genes involved in sperm motility, apoptosis and oocyte maturation were dysregulated between high- and low-fertility bulls. The gene regulation of common transcripts (P ≤ 0.05) and proteins are presented in Supplementary File 4.

**Figure 5:**
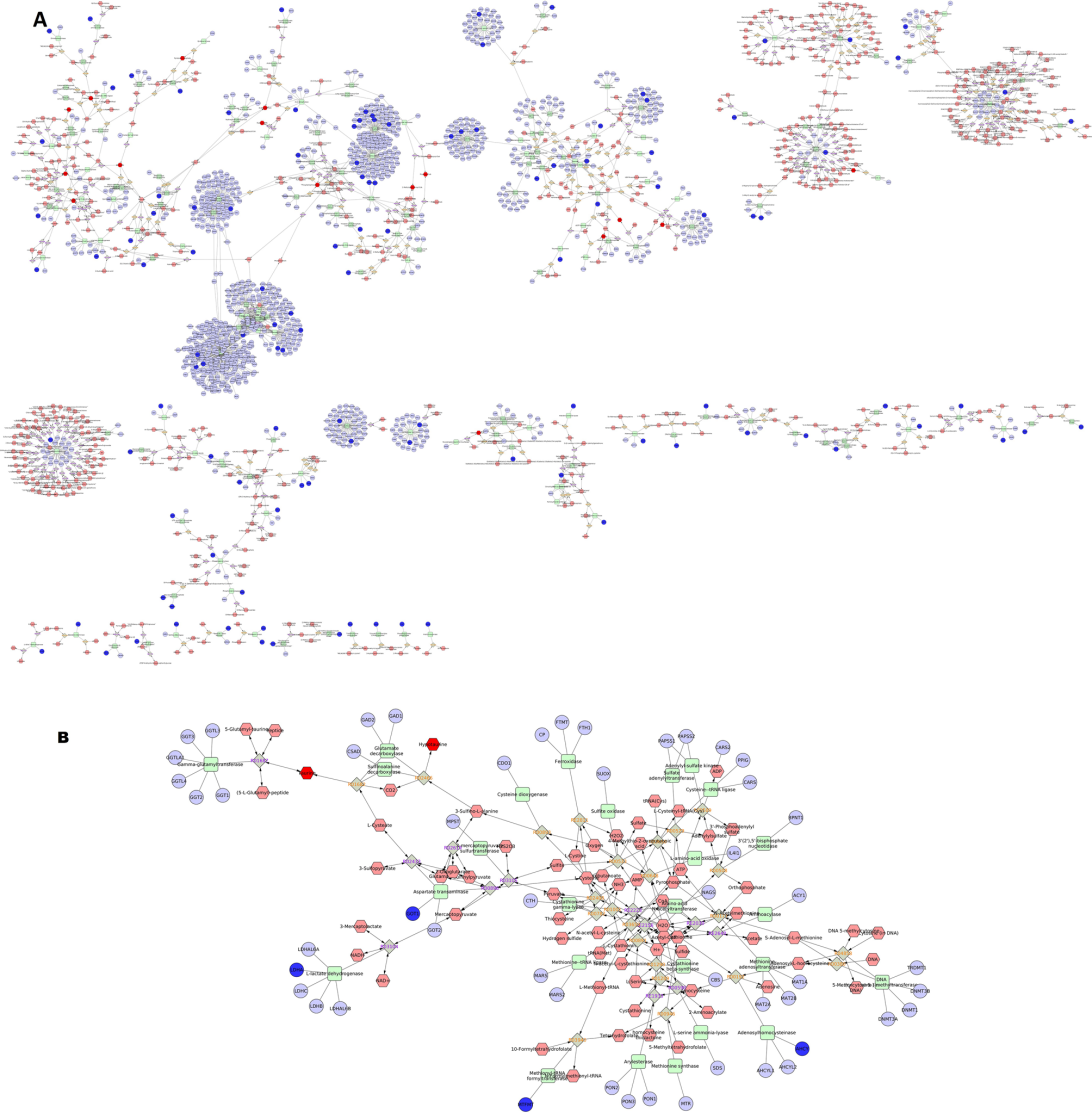
(**A**) Interaction network of dysregulated transcripts and metabolites. (**B**) Subnetwork of Methionine and cysteine metabolism.

An integrated network analysis between the total dysregulated transcripts and metabolites was carried out to find out the interaction and pathways in which transcripts and metabolites were involved (Fig. 5A). Dysregulated transcripts and metabolites both were involved in pathways like Glycolysis and gluconeogenesis metabolism (Fig. 5B), methionine and cysteine metabolism (Fig. 5B), Phosphatidylinositol phosphate metabolism, purine metabolism, pyrimidine metabolism and, Tyrosine metabolism and TCA cycle. Important metabolites like s-maleate, Phosphatidylinositol, dTP, dUTP, Iodide, Taurine and hypotaurine were found to interact with the genes like PFKFB4, IPMK, FOLR1D, DNM2, EEF2, PRDX6 and CARS2 respectively.

**Figure 6:**
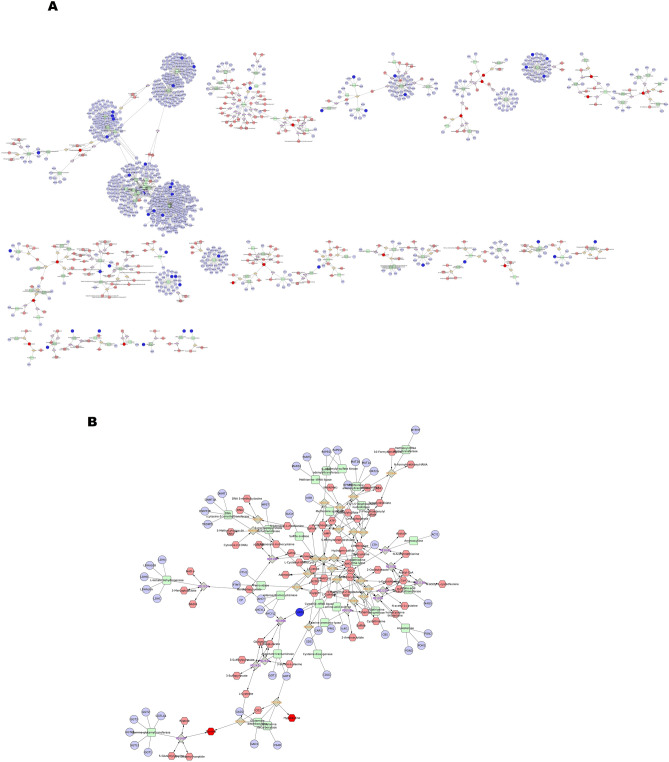
(**A**) Interaction network of dysregulated proteins and metabolites. (**B**) Subnetwork of Methionine and cysteine metabolism.

A protein-metabolite network was generated from the data obtained by the dysregulated proteome and metabolites (Fig. 6A). The network analysis revealed that the proteins and metabolites were commonly involved in different pathways like Glycogen and Gluconeogenesis metabolism, Methionine and cysteine metabolism (Fig. 6B), purine and pyrimidine metabolism and TCA cycle. The results from the Transcriptome-metabolite network and Protein-metabolite network showed that there are common pathways and metabolites exist between the two networks which are mainly co-expressed with the dysregulated transcripts and proteins. All these dysregulated metabolites, proteins and transcripts were found to have their association in sperm motility, regulation of apoptosis, sperm maturation and fertilisation.

**Figure 7:**
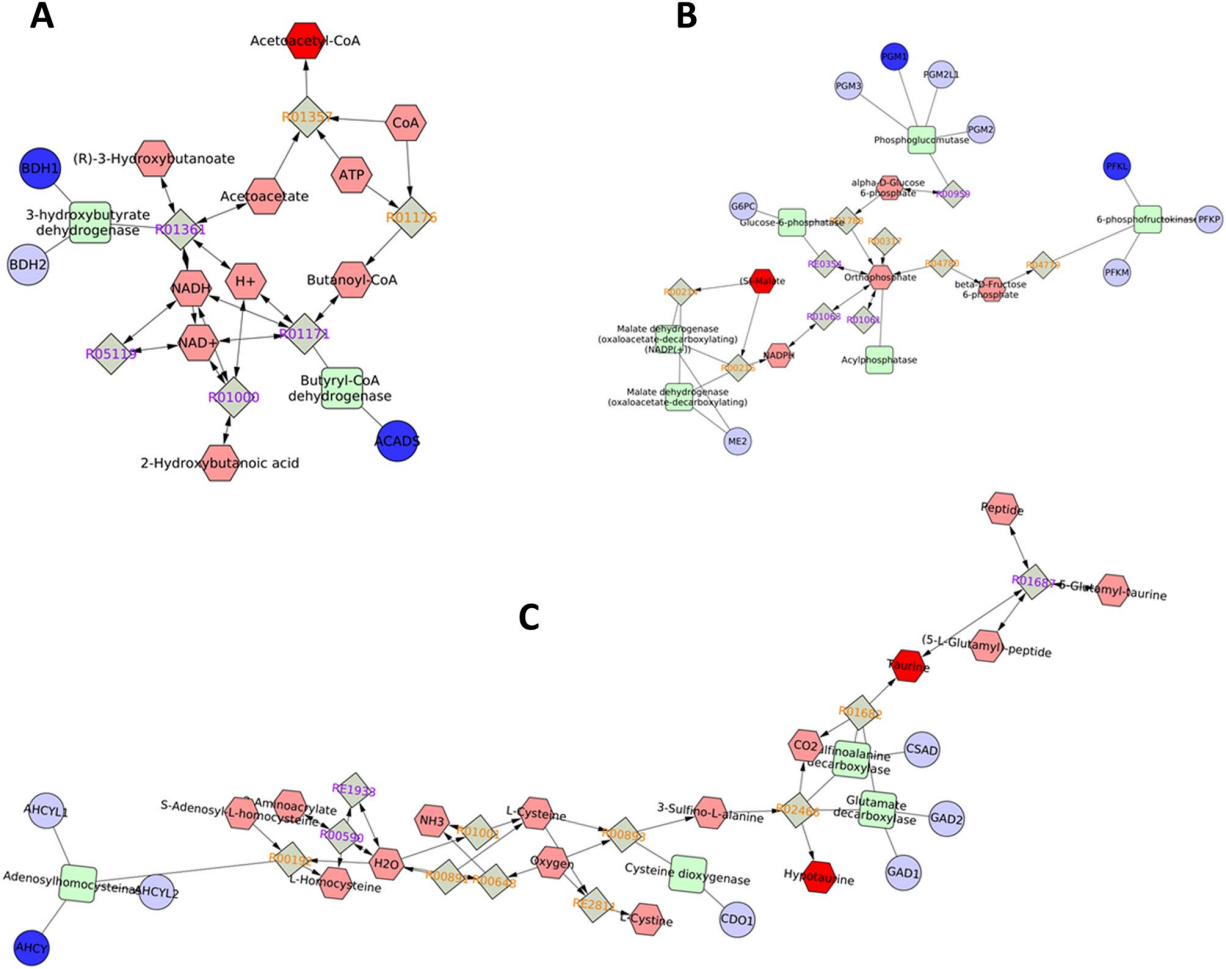
Integrated network of dysregulated transcripts, proteins and metabolites in low-fertility bulls. (**A**) Butanoate metabolism. (**B**) Glycolysis and gluconeogenesis metabolism. (**C**) Methionine and cysteine metabolism.

**Figure 8:**
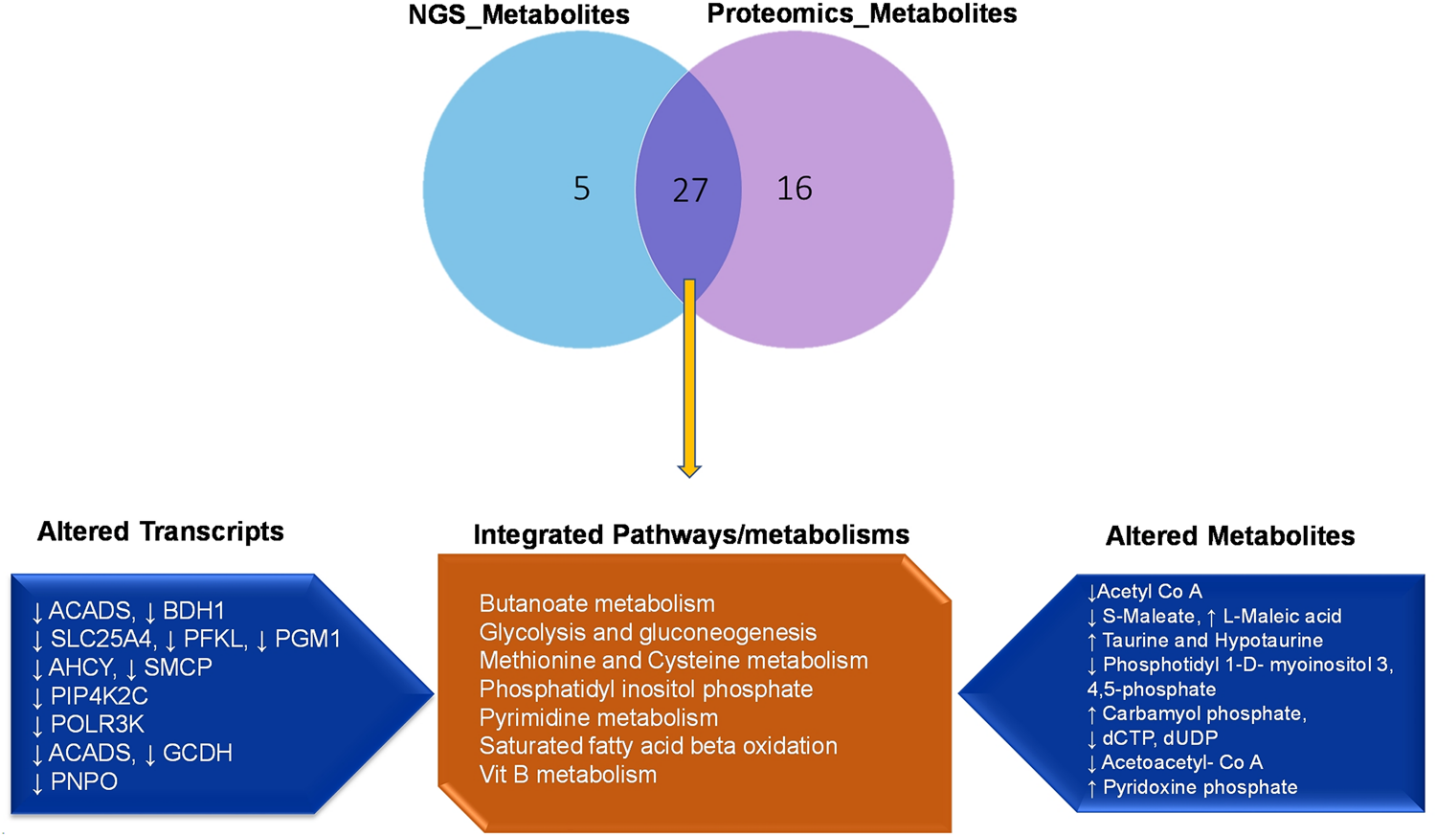
Venn diagram of common dysregulated pathways of translated proteins and metabolites.

KEGG pathway analysis of DEMs and DEPs having corresponding differential expression at transcript level revealed 14 pathways wherein the metabolites and translated proteins were involved. Among these pathways, significant interaction between the transcript, protein and metabolites was observed in seven pathways viz. Butanoate metabolism, Glycolysis and gluconeogenesis, methionine and cysteine metabolism, phosphatidyl inositol phosphate metabolism and purine and pyrimidine metabolism suggesting these pathways are altered or dysregulated in low-fertility bulls (Fig. 7A–C). Further, a network involving DEMs and DEPs having corresponding differential expression at transcript level also was constructed and found that metabolites like Acetoacyl Co-A, S-maleate, Taurine, Hypotaurine, Pyridoxine phosphate, dCTP and dUDP were found to be interacting with the proteins as well as the transcripts (Fig. 8).

### Correlation analysis of functional biomolecules with semen attributes

**Figure 9:**
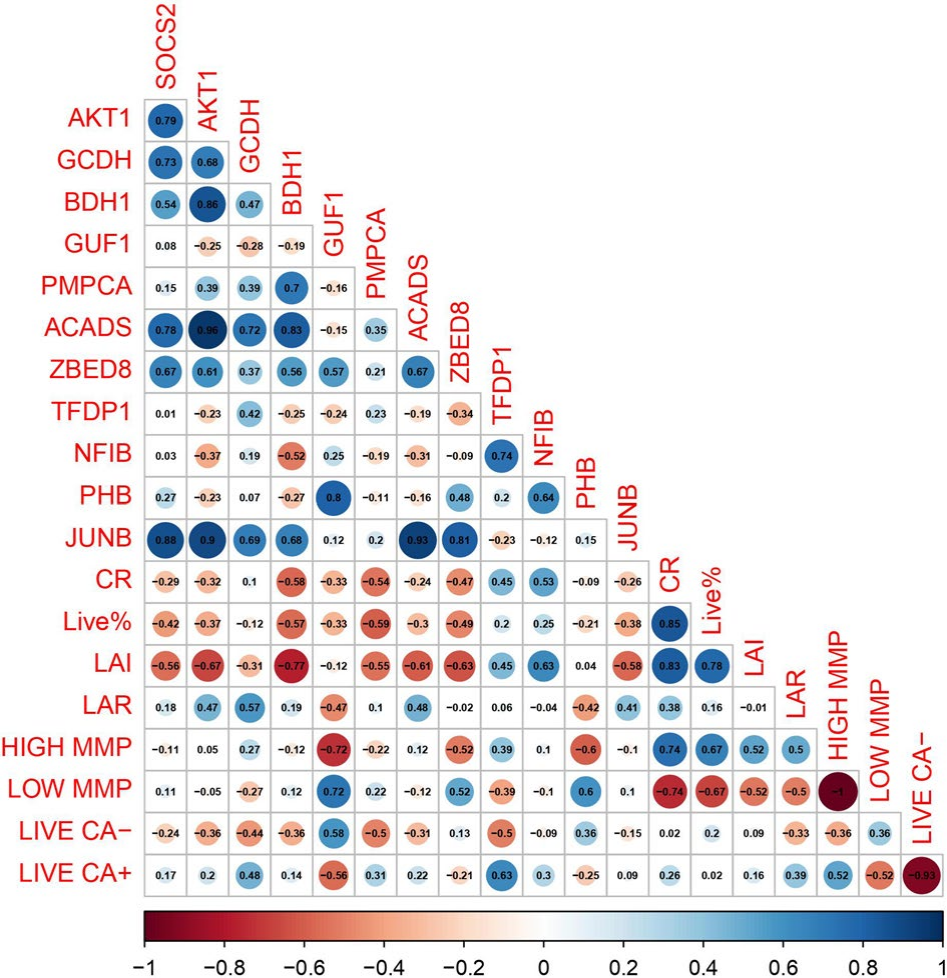
Correlation matrix of sperm functional parameters with selected genes involved in various biological, molecular and cellular components of metabolic pathways.

Correlation analysis was carried out between the translated proteins (commonly dysregulated between transcripts and proteins) and sperm functional parameters (Fig. 9). This analysis revealed that the conception rate (CR) was negatively correlated with genes like SOCS2, AKT1, BDH1, JUNB, ACADS, GUF1, PMPCA and ZBED8. The high MMP and live acrosome intactness of the sperm were found to have negatively correlated with the genes like SOCS2, GUF1, PMPCA, and ZBED8.

## Discussion

Spermatozoa delivers, not just the paternal DNA, but other components such as mRNA, miRNA and transcription factors and other cell signalling molecules, into the oocyte at the time of fertilization^28^. Molecular content of spermatozoon are dynamic, and the factors affecting spermatogenesis and epididymal maturation may influence the sperm composition and hence it is vital to study the molecular details of sperm in order to predict the fertility of bulls^29, 30^. Although the advanced semen evaluation methods like Flow cytometry can aid in analysing several sperm quality parameters in relation to fertility, it may not be sufficient to understand the alterations in the molecular health of spermatozoa in infertile/sub-fertile males with normal spermiogram. Greater time (~ 60 days) is needed for spermatogenesis and final sperm maturation in bulls, and there is ample time for molecular, cellular, and physiological errors to occur that can hamper sperm production and render infertility^24^. Defects in the male germ cells during foetal life may be more probable causes of infertility than defects incurred in later phases of development^24, 27^. Therefore, more comprehensive and complicated studies spanning developmental stages and robust methods are needed to accurately ascertain semen quality and predict bull fertility. There is a need for additional knowledge on the expression levels and functions of sperm transcripts, proteins, and metabolites in relation to fertility, which can elucidate additional fertility markers that can be used in combination for fertility prediction. In this context, application of integrated *omics* or mutli-*omics* helps researchers generate extensive knowledge to better understand the unravelling physiological mechanisms underlying subpar male fertility.

In the current study, we utilized comparative high-throughput transcriptomic, proteomic and metabolomic analysis to identify the molecules and key pathways associated with bull fertility. We identified a total of 18,068 transcripts, 5041 proteins and 3704 metabolites in bull spermatozoa, of which the expression of 4766 transcripts, 785 proteins and 33 metabolites were dysregulated between high- and low-fertility bulls. The number of transcripts, proteins and metabolites observed in the current study is in agreement with those reported earlier^13, 16, 17, 20, 22, 23^.

Among the total dysregulated genes, the top upregulated genes (TPT1, PFN1, ACTG1, RPS11, RPL18A, TMSB10, RPS3, RPL3, EEF2 HSP8 and HSC70) were reported to have their roles in sperm motility, apoptosis, early embryonic development and fertilisation^16, 25, 31^. The top downregulated genes (UPF1, TSSK6, ICAM1, CDC34, MRPL21, FAM57A, AQP7, ANKRD9, TEX264, LUZP1) were reported for their role in protein phosphorylation, sperm maturation, spermatogenesis, gamete fusion, cryotolerance capacity and sperm motility^32–34^. An interesting observation of the current study was that expression of several ribosomal transcripts (RPL5, RPL30, RPL3, RPS8, RPL31, RPL11, RPLP0, RPL8, MRPL21, RPS28, RPL18A, RPL14, RPS3, RPS2, RPS11, RPL19, RPS12) involved in the ribosomal pathway were found to be altered in low-fertility bulls. The presence of ribosomal protein transcripts in spermatozoa and their differential expression between high- and low-fertility bulls indicate their crucial role in the production of healthy sperm and fertility^35^. Defects in ribosome biogenesis could affect the assembly of ribosomes in spermatozoa mitochondria, which further may alter the functions of mitochondria in spermatozoa^16^. Another important finding was that the transcriptional abundance of several genes (COX1, ND1, ND2, ND5, ND4) involved in oxidative phosphorylation pathway were found to be downregulated in the low fertility bulls. Any variations in this pathway will alter the sperm metabolism and function^36–38^ as this pathway is crucial for the sperm to synthesize ATP and produce energy in all mammals^34, 39^. Hence, the impaired oxidative phosphorylation could be the major contributing factor for the low infertility in these bulls.

Various proteins exists in the spermatozoal membrane, acrosome, flagellum, cytoplasm and nucleus play important roles in sperm motility, fertilisation and cell signalling which may be vital in determining the fertility of the bulls^24^. We observed that expression of 785 sperm proteins were dysregulated between high- and low-fertility bulls.

GO and KEGG analysis revealed that sperm proteins involved in various important pathways are downregulated in low-fertility bulls. Important proteins like ATP6V1C1, ATP6V1H, AHCY, GAPDHS, POLR3GL, PFKL, ACADS, and TKTL1 are central to the metabolic pathways and are significantly downregulated in low-fertility bulls. GADPHS (Glyceraldehyde 3-phosphate dehydrogenase-S), a sperm-specific glycolytic enzyme, is reported to be essential for sperm motility and male fertility^40^. TKTL1 was reported to be associated with normal spermatogenesis as it was not detected in the patients suffering from obstructive or non-obstructive azoospermia^41^. AMPK pathway is reported to be key mechanism in regulation of energy metabolism homeostasis^42^, and therefore, it has recently emerged as a new signalling pathway that exerts a necessary control of sperm function^43^. Arginine and proline metabolism, was found to play an important role in energy metabolism, and some substrates could be used in the sperm medium for storage and cryopreservation^44^. The network analysis revealed that the downregulated proteins were associated with many important pathways like execution of phase of apoptosis (ENDOG, CASP6), progesterone mediated oocyte metabolism (CDK2, CDC25C, GNAI1, CCNA2, CDC27, ANAPC10), sperm motility (TEKT1, TEKT4, LRRC6, TEKT3, SLC22A16, IQCF1) and single fertilisation (CCT7, SLC22A16, ABHD2, PRSSS37, ZP4). SLC and TEKT family proteins were reported to play a pivotal role in maintaining the normal flagellar structure, progressive motility and fertility in mouse spermatozoa^45, 46^. CDC25C and CDC27 were observed to be important for phosphorylation^47, 48^ and ZP4 is important for sperm egg fusion and acrosome reaction^49^. All the proteins mentioned in these pathways were observed to be significantly downregulated in low-fertility bull spermatozoa. The top down regulated sperm proteins (TXNDC9, RPL12, POU5F1, TAGLN) in low-fertility bulls are involved in key sperm functions like resistance to oxidative stress^50^, blastocyst formation^51^, energy metabolism, sperm motility^52^ and sperm storage in the female reproductive tract^53^. It has been reported that TXNDC family genes (Txndc2 and Txndc3) deficiency in spermatozoa lead to loss of motility, damage of DNA at higher rates, increased ROS, enhanced formation of lipid aldehyde-protein adducts, and impaired protamination of the sperm chromatin^49^. TXNDC9 was reported to diminish the chaperonin TCP1 complex ATPase activity significantly, thus negatively impacts protein folding, including that of actin or tubulin^54^.

We observed that the dysregulated metabolites were found to be involved significantly in pyrimidine metabolism, taurine and hypotaurine metabolism, propanoate metabolism, synthesis and degradation of ketone bodies, nitrogen metabolism, Vitamin B6 metabolism and Valine, leucine and isoleucine metabolism. Pyrimidine metabolites such as uridine may enhance the sperm motility or act as anti-oxidant^55^ and propanoate metabolism was observed to play an important role in cryopreservation of spermatozoa^56^. The metabolites involved in Taurine and hypotaurine metabolism were reported to be essential for providing energy and antioxidative protection to the spermatozoa for their progressive motility^57^. In low-fertility bulls, significantly dysregulated metabolites included Hypotaurine, Selenocysteine, dITP, Maleic acid, d-cysteine, Acetoacetyl-CoA, Phosphatidylinosito and dUTP. It is well documented that hypotaurine plays a vital role in antioxidant activity. In human sperm, the low concentration of hypotaurine were considered as a marker of sperm stress, suggesting that the oxidation of hypotaurine to taurine may occur in poor quality spermatozoa^58^. Selenocystiene, is reported to be associated with sperm quality while maleic acid is involved in pyruvate oxidation in sperm mitochondria^22^.

Having analysed the individual omics results of spermatozoa between high- and low-fertility bulls, we applied an integrated -*omic* approach and found that 5041 transcripts detected in bull spermatozoa had corresponding proteins also. Integrated transcriptome and proteome network analysis revealed that the translated proteins were found to have relevance in important pathways like positive regulation of cell death (TIMP3, GSN, BCL2A1, APOPT1, SUDS3, ZC3HB,GZMA, BTG1), AMPK signalling pathway (SCD5, PFKB4, PPP2R2B, PFKM and EEF2), sperm motility (TEKT4, SORD, TEKT3, TEKT1, LRRC6, SLC9C1, SLC22A16, and IQCF1), single fertilisation (ABHD2,ZP4,CCT7,CD9,CCT4 and PRSS37), and execution phase of apoptosis (CASP6, INSL3, EPB4IL5 and SPRAC). The overlap analysis between the DEG (383 transcripts) and DEP (785 proteins) resulted in a list of 182 translated proteins. These DEPs were found to be involved in important pathways like metabolic pathway (ACADS, GCDH, and AKT1), TNF signalling pathway (JUNB, ZPR1), Rap1 signalling pathways (FGF5, P2RY1, VEGFB, AKT1, PFN1, MAPK13), Prolactin signalling pathway (SOCS2) and pentose pathway (PFKL, PGM1, TKT1). These molecules are reported to have important functions in sperm quality and fertility. For instance, AKT has been shown to mediate various important process like spermatogenesis, sperm maturation, and fertilization^59^ while low levels of sperm GCDH was reported to be a cause of asthenozoospermia^60^. ZPR and JUNB were observed to be associated with embryonic cleavage, blastocyst formation, blastocyst development and blastocyst growth, placentation and embryogenesis^61–63^. Fibroblast growth factor-5 (FGF5) promotes spermatogonial stem cell proliferation^64, 65^. These proteins are found to be involved in playing vital role in sperm motility, mobilization of acrosomal calcium during sperm exocytosis, regulation of spermatozoal apoptosis and maintenance of acrosome and chromatin integrity. A correlation analysis was also carried out to correlate the important translated proteins (dysregulated) and sperm functional parameters (Table 4). The analysis revealed a negative correlation of conception rate of the bulls with the SOCS2, AKT1, BDH1, ACADS and JUNB. These dysregulated genes in low fertility bulls were also found to have negative correlation with the mitochondrial membrane potential and live acrosome intact of the spermatozoa. The negative correlation of these dysregulated genes with the important sperm functional parameters and conception rate mirrors the cause for low fertility in bulls.

**Table 4:** List of selected dysregulated genes used for correlating with in sperm functionality.

Gene name	Transcript	Protein
SOCS2	− 1.43	0.95
AKT1	1.17	0.72
GCDH	− 2.46	0.96
BDH1	− 1.64	1.68
GUF1	1.11	− 0.98
PMPCA	− 3.18	0.73
ACADS	1.27	0.88
ZBED8	− 1.09	0.95
TFDP1	− 1.33	− 0.75
NFIB	− 1.08	− 1.04
PHB	− 2.11	0.83
JUNB	3.87	0.73

Upon integrating the transcriptome-metabolites and protein-metabolites, we studied their interaction in important pathways associated with sperm quality and fertility. The analysis revealed that Androgen and oestrogen biosynthesis metabolism, Galactose metabolism, TCA cycle, Propanoate metabolism, Purine metabolism and Glycolysis and gluconeogenesis metabolism pathways were to both the integrations. Glycolysis and gluconeogenesis consists of a series of biochemical reactions and metabolites to generate energy in the form of ATP^37^. It was reported that maintenance of intracellular energy status is essential for sperm function^7^, however, we observed that the levels of glycolytic metabolites were found to be significantly downregulated in low-fertility bulls. Pyruvate metabolism is crucial for understanding the contributions of OxPhos for the fertilizing capacity of spermatozoa. Sperm mitochondria produce ATP through aerobic oxidation, in which the tricarboxylic acid (TCA) cycle in the mitochondrial matrix is cardinal^66^. TCA cycle is known to be a crucial metabolic pathway that contributes to production of ATP^67^. These pathways are very important because of their vital role in sperm metabolism, sperm motility and in apoptotic mechanisms.

We further integrated the transcriptome, proteome and metabolite data to find the interaction among them. We found the interaction of transcript, protein and metabolite in seven metabolic pathways, which include Butanoate metabolism, Glycolysis and gluconeogenesis, Methionine and cysteine metabolism, Phosphatidyl inositol phosphate, pyrimidine metabolism, saturated fatty acid beta oxidation and Vit B metabolism. Methionine and cysteine metabolism was significant, and the observed metabolites were taurine and hypotaurine, which plays a significant role in maintaining sperm motility, capacitation and the acrosome reaction^57^. Butanoate metabolism, glycolysis and gluconeogenesis are crucial for sperm motility and energy production in spermatozoa^68, 69^. Glycolysis and gluconeogenesis pathways are very vital for sperm motility as glycolysis could produce ATP adjacent to the site it is required to support active sliding of the flagellar filaments^69^. Various genes, proteins and metabolites found in these pathways and their function are listed in the Table 5. In low-fertility bulls, downregulated sperm proteins like ACADS (Acetyl Co-A), PFKL, PGM1 (S-Maleate), AHCY (Taurine and Hypotaurine), POLR3K (carbamoyl phosphate), ACADS and GCDH (Acetoacetyl Co-A) were significantly had significant interaction with metabolites. ACADS was reported to be responsible for β-oxidation in the mitochondria of cells and its dysregulation or downregulation leads to decreased ability to oxidize fatty acids, thereby signifying sperm metabolic dysfunction. PFKL is associated with capacitation and acrosome reaction in boar spermatozoa^70^. AHCY is reported to be involved in the energetic metabolic pathway, leading to the production of phosphocreatine, which is essential for carp sperm motility^71^, while GCDH was reported to be associated with progressive motility in human beings^60^. From the foregoing information, it is very much evident that the results of integrated multi-omics analysis of bull spermatozoa link up sperm metabolism with bull fertility and it is clearly appreciable that the expression of molecules associated with sperm metabolism was altered in low-fertility bulls.

**Table 5:** The integrated multi-omics data analysis revealed genes, proteins and metabolites that potentially correlate with bull fertility.

Pathway ID	KEGG pathway	Gene/translated protein	Significant function	Interacting metabolites
bta00650	Butanoate metabolism	ACADS, BDH1	Sperm metabolism	Acetyl Co-A
bta00010	Glycolysis and Gluconeogenesis	SLC25A4, PFKL, PGM1	Negative regulation of Apoptosis in sperm	S-maleate, l-Maleic acid
bta00270	Methionine and Cysteine Metabolism	AHCY, SMCP	Sperm motility, Oxidative phosphorylation	Taurine and hypotaurine
bta04070	Phosphatidyl inositol phosphate	PIP4K2C	Sperm motility and Acrosome reaction	Inositol-3.4, phosphate phosphotidyl 1-d-myoinositol
bta00240	Pyrimidine metabolism	POLR3K	Not identified	Carbamyol phosphate, dCTP, dUDP
bta01212	Saturated fatty acid beta oxidation	ACADS, GCDH	Sperm motility	Acetoacyl Co-A, acetyl Co-A
bta00750	Vit B metabolism	PNPO	Not identified	Pyridoxine phosphate

It may be concluded that integrated multi-omics analysis of spermatozoa from high- and low-fertility bulls revealed that molecules and pathways associated with Glycolysis and gluconeogenesis, cysteine and methionine metabolism and butanoate metabolism were dysregulated in low-fertility bulls. These molecules and pathways are important for sperm metabolism, energy production and regulation of oxidative phosphorylation. All these findings collectively indicate that molecules governing sperm metabolism potentially influence bull fertility. Therefore, there is a need for an in-depth analysis of the molecules involved in sperm metabolic pathways to expand our understanding on bull fertility and to find out fertility markers for selection of bulls for high fertility.

## Materials and methods

### Ethics statement

The present study was carried out at the Theriogenology Laboratory, Southern Regional Station of ICAR-National Dairy Research Institute, Bengaluru, India. All the experiments were conducted in accordance with the guidelines and regulations laid down and duly approved by Institute Animal Ethics Committee (CPCSEA/IAEC/LA/SRS-ICAR-NDRI-2019/No.04).

### Animals and sample collection

Holstein Friesian crossbred bulls (n = 50) that qualified the breeding soundness examination and regularly used for artificial breeding were utilized for the study. All the experimental bulls have qualified the breeding soundness evaluation and were routinely used for semen collection and artificial breeding. The bulls used in the current study were maintained under tropical climate following standard bull management practices as per the Minimum Standard Protocol for semen production. The bulls were fed with concentrate ration containing 21% crude protein and 70% total digestible nutrients along with ad libitum seasonal green fodders. The bulls had free access to clean drinking water throughout the day. Vaccination, de-worming, regular check-up for communicable diseases and other herd-health programmes were followed as per the farm schedule, to protect the animals from diseases and to produce quality semen. As per the standard method, semen from these bulls were collected using artificial vagina and cryopreserved in Tris-Egg Yolk-Citrate extender using a programmable freezer. The motility of the semen from these bulls were assessed by a single qualified technician immediately after the collection and after freezing using microscopy. The fertility of the bulls were calculated based on conception rates (CR). The mean number of artificial inseminations performed per bull for calculation of conception rates was 1351 (range 841–2202). The CR of the bulls were calculated based on the number of animals conceived out of total number of animals inseminated (up to 3 inseminations). Artificial inseminations (AI) were carried out by certified AI workers in a particular region where the external factors influencing the success of AI are almost similar for all animals including the management practices. Further the effect of non-genetic factors on conception rate was adjusted as previously described^10^. The adjusted conception rate was used for the calculation of bull fertility. Bulls with conception rate more than Mean + 1 standard deviation (3.95%) were considered as high-fertility and those bulls with conception rate less than Mean − 1 standard deviation were considered as low-fertility. The differences in conception rates between high- and low-fertility bulls were more than 7.9%. The conception rates and number inseminations done per bull are presented in the Supplementary Fig. 5. Cryopreserved semen samples from six high-fertility and six low-fertility bulls (three ejaculates pooled for each bull) were used for analyses. The semen samples from both the groups were subjected for purification, isolation of RNA, cDNA synthesis, protein and metabolite extraction as described in the following headings.

### Flow cytometry assessment of seminal parameters

Sperm membrane integrity, acrosome integrity, mitochondrial membrane potential and intra cellular calcium levels were assessed in high and low fertility using a Flow Cytometer (CytoFLEX S, Beckman Coulter Life Sciences, Indianapolis, IN). Excitation was induced by a blue laser (488 nm). FITC fluorescence was detected with a fluorescence channel (FL) 1 band-pass filter (525/40 nm) and PI fluorescence was measured using a FL3 band-pass filter (585/42 nm). The results of Flow Cytometry analysis are given in Supplementary Fig. 1.

### Membrane integrity

SYBR-14 (Invitrogen, Thermo Fisher scientific, USA) and Propidium iodide (PI) (Invitrogen, Thermo Fisher scientific, USA) was used for assessing the sperm membrane integrity as previously described^12^. SYBR-14 working solution (1 mM; 1.2 mL) was added to 2 million spermatozoa in 200 mL of sp TALP and incubated at 37 °C for 10 min in the dark and then 2 mL of PI (2.4 mM) was added and further incubated for 2 min before analysing in flow cytometer.

### Acrosomal integrity

Acrosomal integrity was assessed using FITC-PNA (fluorochrome fluorescein isothiocyanate-Peanut agglutinin) and PI combination, 1 µL of 1 mg/mL FITC-PNA was added to approximately 2 million spermatozoa and incubated at 37 °C for 10 min, then 1 µL of 2.4 mM PI was then added and incubated for 2 min and assessed using flowcytometry.

### Mitochondrial membrane potential

Mitochondrial membrane potential was assessed using JC-1 stain. Approximately to 2 million spermatozoa, 5 µL of JC-1 (0.2 mM) was added and incubated at 37 °C for 30 min before analysing in flow cytometer.

### Intracellular calcium

Intracellular calcium assessment in spermatozoa were carried out using Fluo 3 AM (Invitrogen, ThermoFischer scientific, USA). To 2 million spermatozoa, 3 µL of Fluo 3 AM (1 mM) was added and incubated at 37 °C for 30 min. PI (2 µL) was then added and further incubated for 2 min before analysing in flow cytometer.

### Sperm transcriptomic analysis

#### Sperm purification, RNA isolation and cDNA synthesis

Spermatozoa in semen samples were purified using Percoll gradient (90–45% discontinuous) centrifugation to eliminate the semen extender, cryoprotectant, egg yolk particles and epithelial cell contamination^16^. Briefly, the thawed semen (37 °C for 30 s) was carefully layered over the top of prepared Percoll gradient and was centrifuged for 15 min at 950×*g* to obtain the sperm pellet. The semen pellet was washed with phosphate buffer saline (PBS; pH 7.2) three times and subjected to RNA isolation. Total RNA was isolated from Percoll-selected spermatozoa using TRIzol (Ambion, Thermo Fisher Scientific, United States) as previously described by Prakash et al.^16^. The isolated RNA was treated with DNase to eliminate traces of gDNA. The RNA quantification was done using NanoDrop (ND-1000, Thermo Fisher Scientific, United States). Samples with optimal purity (OD 260/280 > 1.8 and 50 ng/mL) were selected for further cDNA synthesis using RevertAid First Strand cDNA Synthesis Kit (Thermo Fisher Scientific, United States, Catalog number K1622) as per the manufacturer’s instructions. Two representative cDNA samples were made from the six high- fertility (HF) bulls by pooling equal quantities of cDNA from three HF bulls each. Similar method was adopted for low-fertility (LF) bulls. The samples were sequenced using Illumina Nextseq-500 sequencing system (Sandor^®^ Lifesciences Pvt. Ltd. Banjara Hills, Hyderabad, India).

#### RNA sequencing and data analysis

The raw data generated from sequencing was analysed using the cloud server *UseGalaxy*^72^. The sequencing library generated Paired-end (76 bp × 2) *fastQ* file Raw data for the 4 (2 high- and 2 low-fertility groups) samples. *FastQC* v0.11.5 was used for checking the quality and GC content of the data generated^73^. Low quality bases were removed and high quality reads were retained with minimum length of 15^74^ based on the *phred* quality score. The removal of low quality bases along with adaptor removal was done using *Cutadapt*^74^. For reference based transcriptomics study, the processed high quality data was aligned against the *Bos taurus* UMD 3.1 genome using *Hisat2 v2.1.0*^75^. After the alignment, aligned files were sorted with the aid of *Samtools* v1.11^76^. Upon alignment, read count was obtained for each transcript in *gff* reference file through *htseq-count* v0.9.1^77^. Sample groups with the read count were further analysed for differential expression using *DESeq*^78^. Expression changes are seen as log2Fold Change, for upregulated transcripts log2FoldChange > 1 while for downregulated transcripts log2FoldChange < − 1. The raw data for the study is available in the NCBI SRA database under project submission ID-PRJNA516089 (https://www.ncbi.nlm.nih.gov/sra/PRJNA516089).

### Sperm proteomic analysis

#### Sperm protein extraction

Purification of the spermatozoa was done using Percoll gradient (90–45% discontinuous) as described above. Protein was extracted from the spermatozoa as per the methods adopted previously by Aslam et al.^2^. For in solution digestion, 100 µg of the sample was taken and diluted with 50 mM Ammonium Bicarbonate before being used for peptide extraction. This sample was treated with 100 mM DTT at 95 °C for 1 h followed by 250 mM IDA at room temperature in dark for 45 min. Then, digested with Trypsin and incubated overnight at 37 °C. The digested peptides were speed vacuumed and reconstituted in 50 µL of water containing 0.1% formic acid. Following centrifugation at 10,000×*g*, 10 µL of the sample supernatant was injected onto a C18 UPLC column for peptide separation. Following digestion and column separation, the sample was run on an LC–MS–QTOF instrument (Waters-Synapt G2), for obtaining the high-resolution data^79^. The samples were administered in triplicate to ensure accurate data collection from each sample and statistical significance. Waters’ MASS LYNX 4.1 program was used to capture and analyze the raw data. The peptides were loaded with buffer A and eluted with buffer B (95% acetonitrile, 0.1% formic acid) at a flow rate of 0.3 mL/min. Using the PLGS program 3.0.2, the individual peptides’ MS/MS spectra was matched to the index, taking into account the mass changes caused by various modifications (Carbamidomethylation (C), Oxidation (M), and so on) (Protein Lynx Global Server)^80^. Following the protein identification of all samples, expression analysis was conducted pair-wise for the samples based on conditions i.e. high-fertility *vs* low-fertility. The spectral data was auto-normalized and analysed by the ProteinLynx Global server (PLGS) application, for measuring the ratios to assess the up-regulation and down-regulation of common proteins. The parameters used for identification are Peptide Mass Tolerance at MS1 level: 50 ppm and Fragment Mass Tolerance at MS2 level: 100 ppm. Carbamidomethyl on cysteine as fixed modification and oxidation of methionine and N-terminal acetylation were considered as variable modifications for database search. The Score is calculated by the expression analysis extension of PLGS Software based on the relevance of the protein present in both the samples being compared. Differentially expressed proteins were identified by calculating fold change of expression values (log base2) with respect to control samples. High fertility bulls samples were taken in as control and Low Fertility were considered as treated. Differentially expressed proteins include upregulated (> twofold) and downregulated proteins (< 0.5-fold) in the treated sample. The unique Proteins mentioned for each Control and Treated are the proteins that did not have any matching peptides or m/z values between the groups.

### Sperm metabolomic analysis

#### Sperm metabolite extraction

The extraction of metabolites from the spermatozoa was carried out as per the methods previously described by Saraf et al.^22^. Briefly, 100 μg of protein equivalent samples were taken for the extraction of metabolites. Pooled samples was added with LC/MS grade water (Merck), Methanol, and acetonitrile (JT Baker, Phillipsburg, NJ) in the ratio of 1:2:2 (i.e. v/v/v). The samples were vortexed for 1 min and then subjected to sonication for 10 min and centrifuged at 12,000×*g* for 15 min at 4 °C. The supernatant was collected and further processed for drying in speedVac (Thermo Fisher Scientific) for 1 h and then processed further for LC–MS/MS analysis.

#### Metabolomic analysis using LC–MS/MS

The LC–MS/MS analysis was performed on a QTRAP 6500 mass spectrometer (SCIEX, Framingham, MA) coupled with 1290 infinity II liquid chromatography system (Agilent Technologies). The samples were then injected on to C18 Zorbax rapid resolution high definition analytical columns (20 × 150 mm, 1.8 μm particle size) with the help of programmable auto sampler. Analyst software version 1.6.3 with the analyst device driver was used to set the parameters for the analysis. The separation of the metabolites was carried out using a 30 min LC method. Solvent A (0.1% formic acid in MilliQ water) and solvent B (0.1% formic acid in 90% acetonitrile) were used for analysis with a standard flow rate of 0.3 mL/min. Elution of metabolites was carried out using the following gradient: 2% B for t = 0 min; 30% B fort = 10 min; 60% B fort = 17 min; 95% B for t = 22–26 min, and 2% B for t = 27.5–30 min. The mass spectrometry data acquisition was carried out with information dependent acquisition (IDA) method, built with enhanced mass spectra (EMS) and enhanced product ion (EPI) i.e. EMS‐IDA‐EPI method in low mass mode. The top five spectra from the EMS mode were used for analysis in the EPI (MS/MS) mode; using high energy collisionally induced dissociation (CID). The metabolite data were acquired in both positive and negative polarities at 4500 V and − 4500 V, respectively, with a probe temperature of 450 °C. The compound parameters were set at a declustering potential of 75 V and collision energy of 45 V. The data was acquired in triplicates. After sorting out the metabolite IDs, the majority of the identified endogenous metabolites are categorized into lipid, phospholipid, glycerophospholipid, amines, common amino acid, fats, and common fatty acid based on functional enrichment of chemical compound in MBrole 2.0 (http://csbg.cnb.csic.es/mbrole2). Antibiotics, pesticides, and heavy metals were also identified as exogenous metabolites in MBrole 2.0, which were excluded from further downstream analysis. Regardless of its polarities, sorted endogenous metabolites of both groups were consolidated to carry out the statistical analysis and enrichment of metabolic pathways in MetaboAnalyst software.

#### Gene ontology and functional pathway analysis

Gene ontology (GO) classification of total dysregulated sperm transcripts, proteins and metabolites were carried out done using Uniprot and the Database for Annotation, Visualization, and Integrated Discovery (DAVID) Bioinformatics Resources (v6.8) and categorized as biological process (BP), cellular component (CC) and molecular function (MF) and Kyoto Encyclopedia of Genes and Genomes (KEGG) pathway. A donut pie chart was plotted for top ten dysregulated BPs, MFs, and CCs using Highcharts. Significantly differentially expressed transcripts, proteins and metabolites with a P-value cut-off of 0.05 were taken in DAVID (Dennis et al.), an online annotation software, and pathway enrichment was carried using *clusterProfiler*^81^. Interaction network analysis of combined GO categories and pathway analysis among the transcripts, proteins, and metabolites was performed using *ClueGo* (Version 2.5.4) and *Cluepedia* (Version 1.5.4) plugins in the open source *Cytoscape* (Version 3.7.1) platform^82^. A network of interactions between all dysregulated transcripts, proteins and metabolites were obtained in the form of different layouts. All bioinformatic analyses were performed by considering *B. taurus* as background species.

#### System biology analysis

For understanding the biological system, a holistic approach where transcriptomics, proteomics and metabolomics data were considered to find the correct biological interpretation (Supplementary Fig. 6). Venn diagram was generated illustrating the overlap between all dysregulated and significant transcripts and all proteins observed in the current study. To know the deciding biological factor, overlap between differentially expressed genes (DEGs) and differentially expressed proteins (DEPs) was acquired. For the associated overlapping gene GO and pathway analysis were carried. To integrate the differentially expressed metabolites (DEMs) with the transcriptomics and proteomics *Metscape v3.1.3.*^83^ plugin was used in *Cystoscope* v3.8.2 tool. DEMs together with DEGs and DEMs with DEPs were analysed together using the *Metscape* and *Metaboanalyst 5.0*. Based on the compound and genes, pathway based analysis was carried for integrating the metabolites with genes and proteins.

### Correlation analysis of functional biomolecules with semen attributes

To understand the relation between the biomolecules (transcript/protein/metabolite) and various semen parameters, correlation analyses was carried out. Commonly dysregulated genes (n = 182) between transcripts and proteins were functionally annotated. The list of dysregulated genes provides variability in the data and distinguishing factor between the sample group (HF and LF samples). Some of these genes involved in biological processes, molecular function and cellular component (negative regulation of protein kinase activity and RNA polymerase II transcription factor activity and localisation in mitochondrial matrix) were correlated with sperm-function attributes (Conception rate (CR), Live%, Live acrosome intact (LAI), Live acrosome reacted (LAR), high mitochondrial membrane potential (high MMP), low mitochondrial membrane potential (Low MMP), Live Calcium negative (Live CA−), and Live Calcium positive (LIVE CA+). The abundance of protein (intensity values) was taken as input to correlate with sperm-function attributes. The R library “corrplot” was used to correlate and generate the visualization.

### Statistical analysis

The descriptive analysis of the conception rate calculated was done using SPSS version 22 (IBM, USA). The division of bulls into high and low fertility was based on mean ± 1 SD. For the bioinformatic analysis the following statistical procedures were adopted Functional enrichment analyses of gene ontology terms and KEGG pathway terms were carried using DAVID. Based on the input list of genes and genes involved in a particular pathway Fisher Exact P value was used to calculate the EASE score, which provides a significantly enriched term. The EASE score cut-off is given by P-value < 0.01. So the terms whose P-values are less than 0.01 were obtained as the enriched term after enrichment analysis. So lesser the P-value, more is the enriched term. The minimum count of genes considered for the term association is ≥ 2. False discovery rate (FDR) is the corrected P-value obtained by Multiple corrections using Benjamini and Bonferroni testing.

## Supplementary Information

Supplementary Figures.

Supplementary Information 1.

Supplementary Information 2.

Supplementary Information 3.

Supplementary Information 4.

Supplementary Information 5.
