# Supplementary material for: Integrated multi-omics analyses reveals molecules governing sperm metabolism potentially influence bull fertility
Source: Sci Rep. 2022 Jun 23;12:10692. doi: 10.1038/s41598-022-14589-w (PMC9226030; doi:10.1038/s41598-022-14589-w)
Supplement: Supplementary file 1 — Supplementary Figures. [file 41598_2022_14589_MOESM1_ESM.docx]

**SUPPLEMENTARY FIGURES**


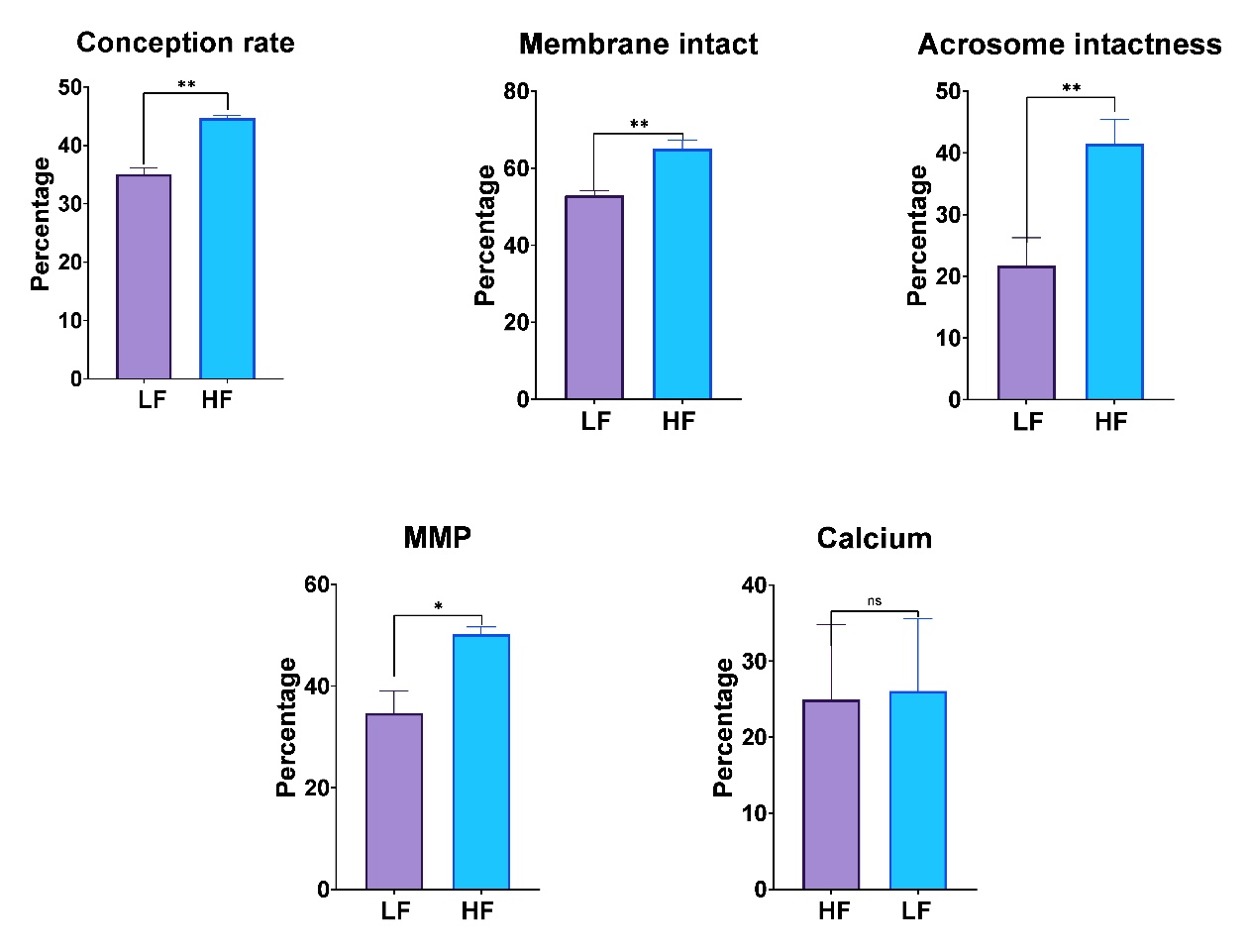


**Fig 1.** Seminal parameters assessed in the frozen semen of high-fertility and low-fertility bulls (six replicates per bull were assessed for Flowcytometry data generation). (LF: Low fertility bulls; HF: High Fertility bulls; MMP: Mitochondrial membrane potential)


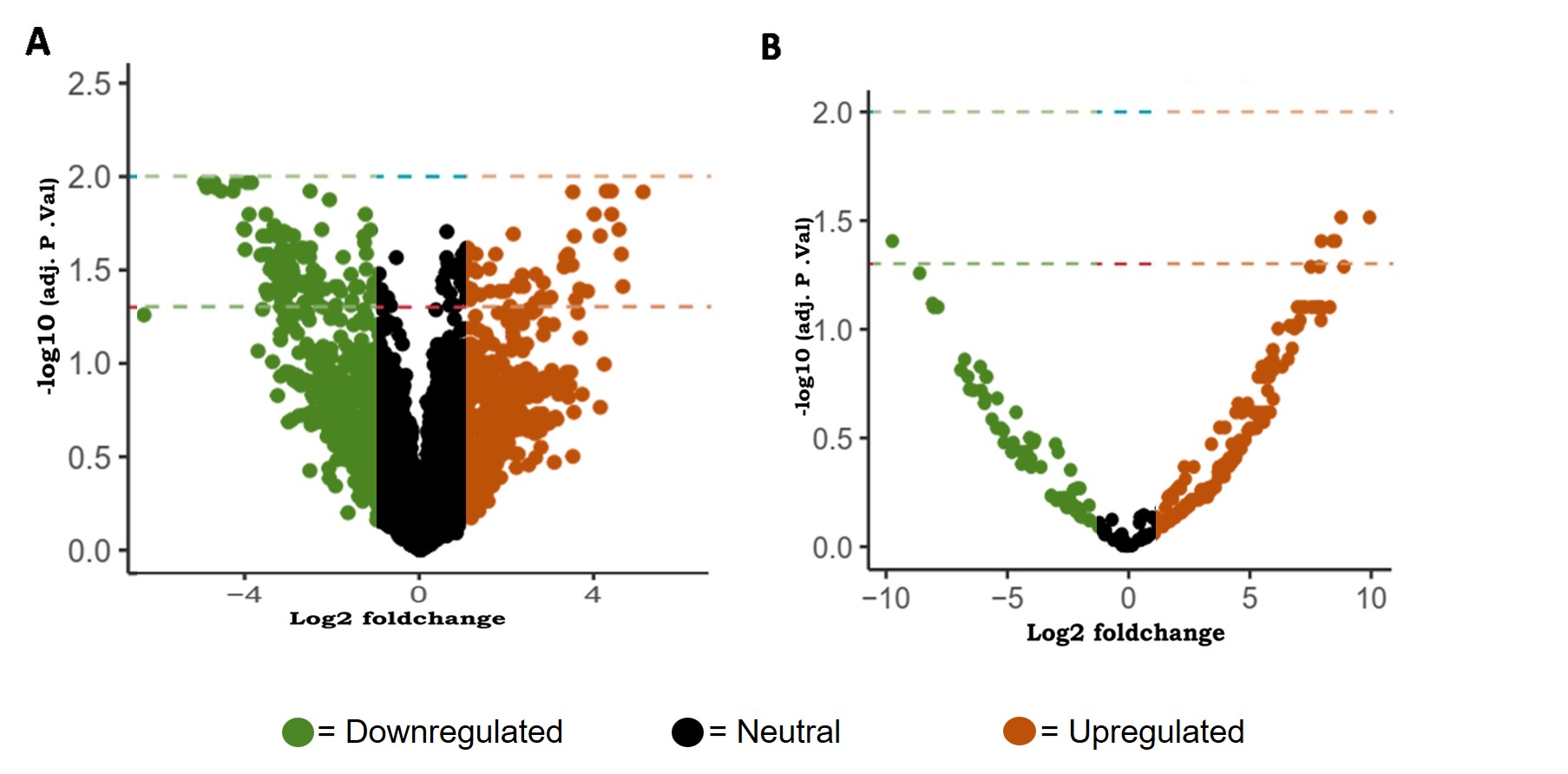


**Fig 2.**  Volcano plot comparison of A) transcripts and B) proteins of high fertility and low fertility bulls.


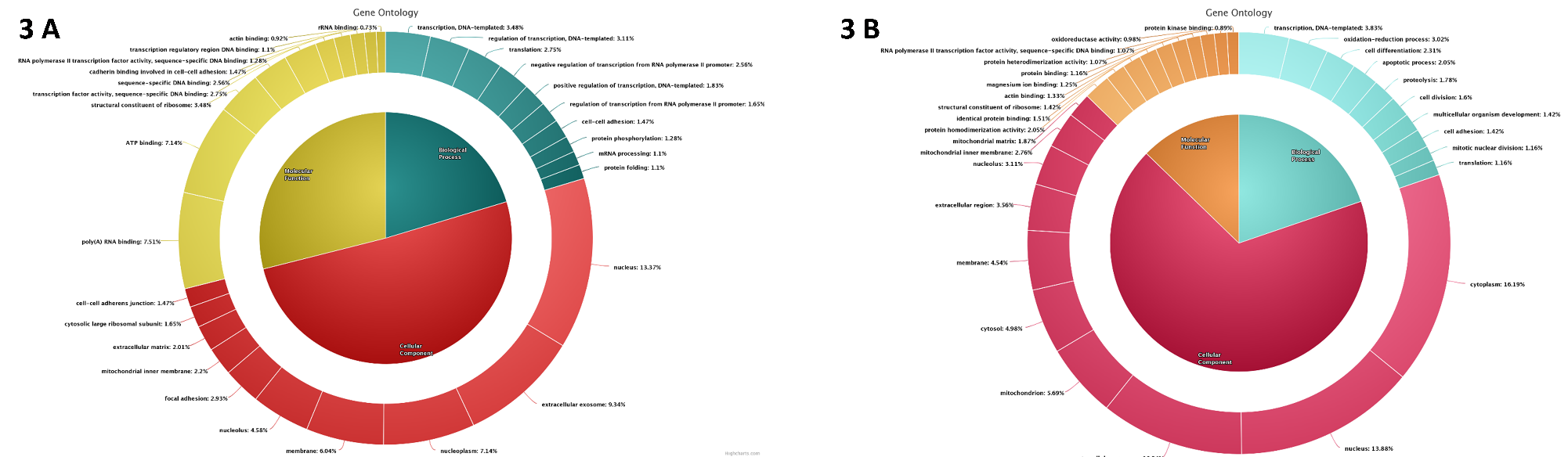


**B**

**A**

**Fig 3.** Top 10 gene ontology categories of A. transcripts dysregulated in low-fertility bulls B. proteins dysregulated in low-fertility bulls.


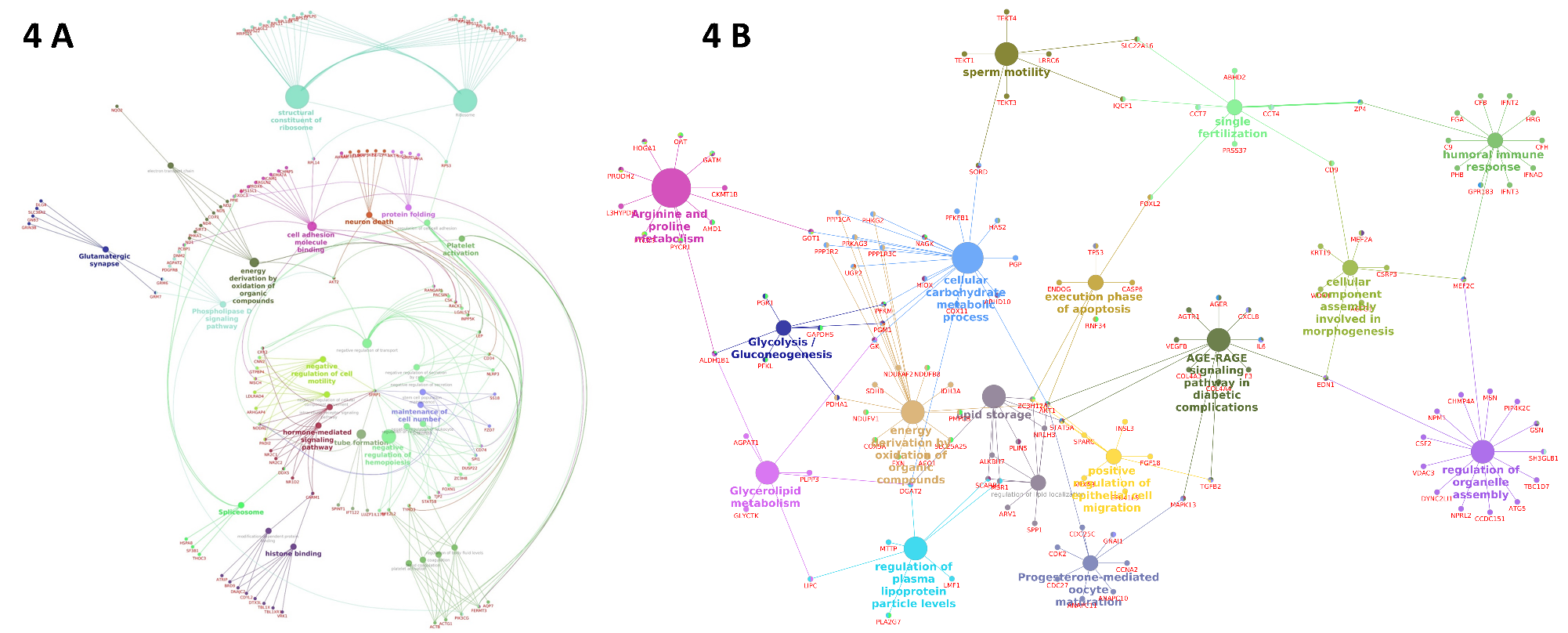


**B**

**A**

**Fig 4.** Network analysis of A. Dysregulated transcripts and B. Dysregulated proteins


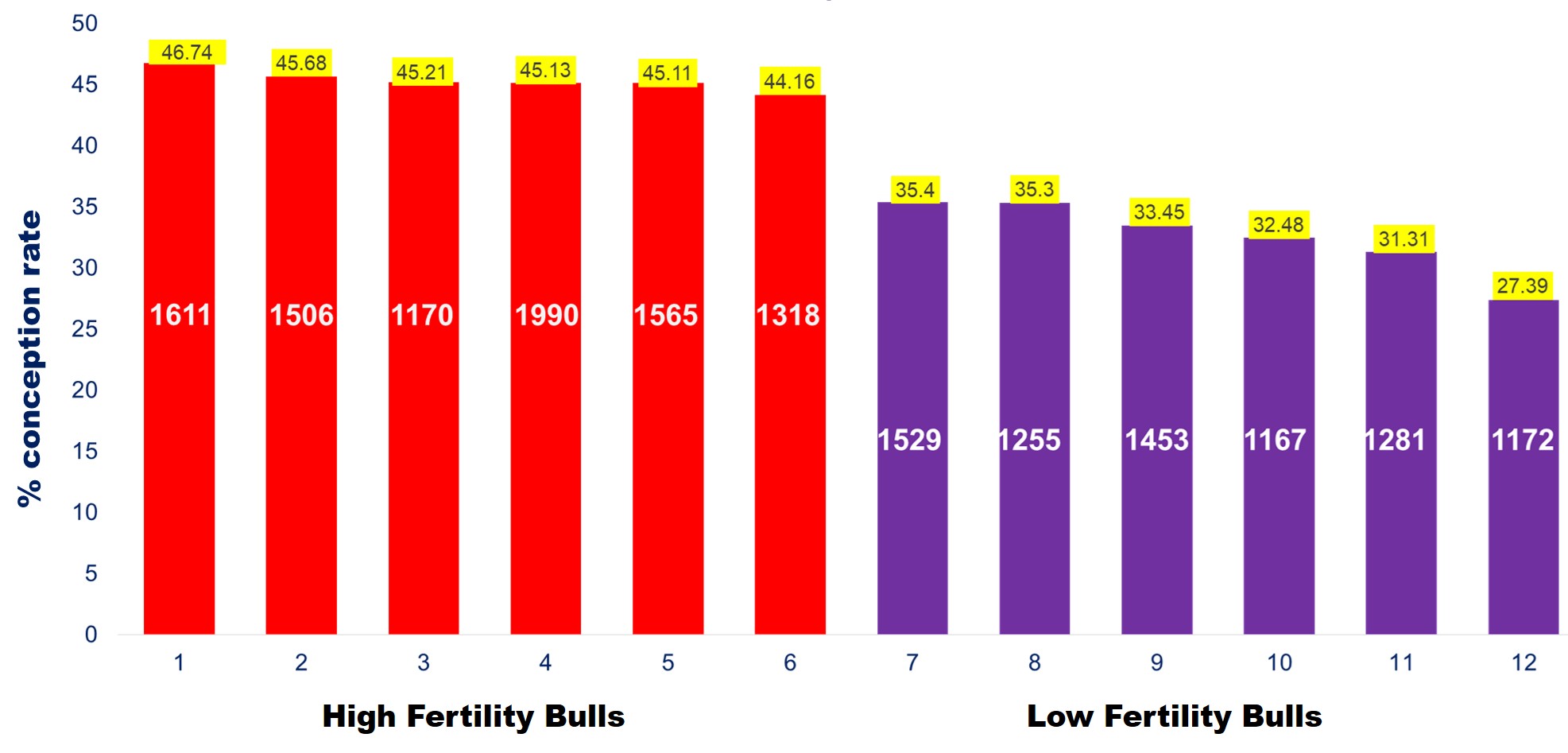


**Fig 5**. The conception rate (above the bar in yellow boxes) and number of inseminations (number with in the bar) of the high- and low- fertility bulls used in the current study.


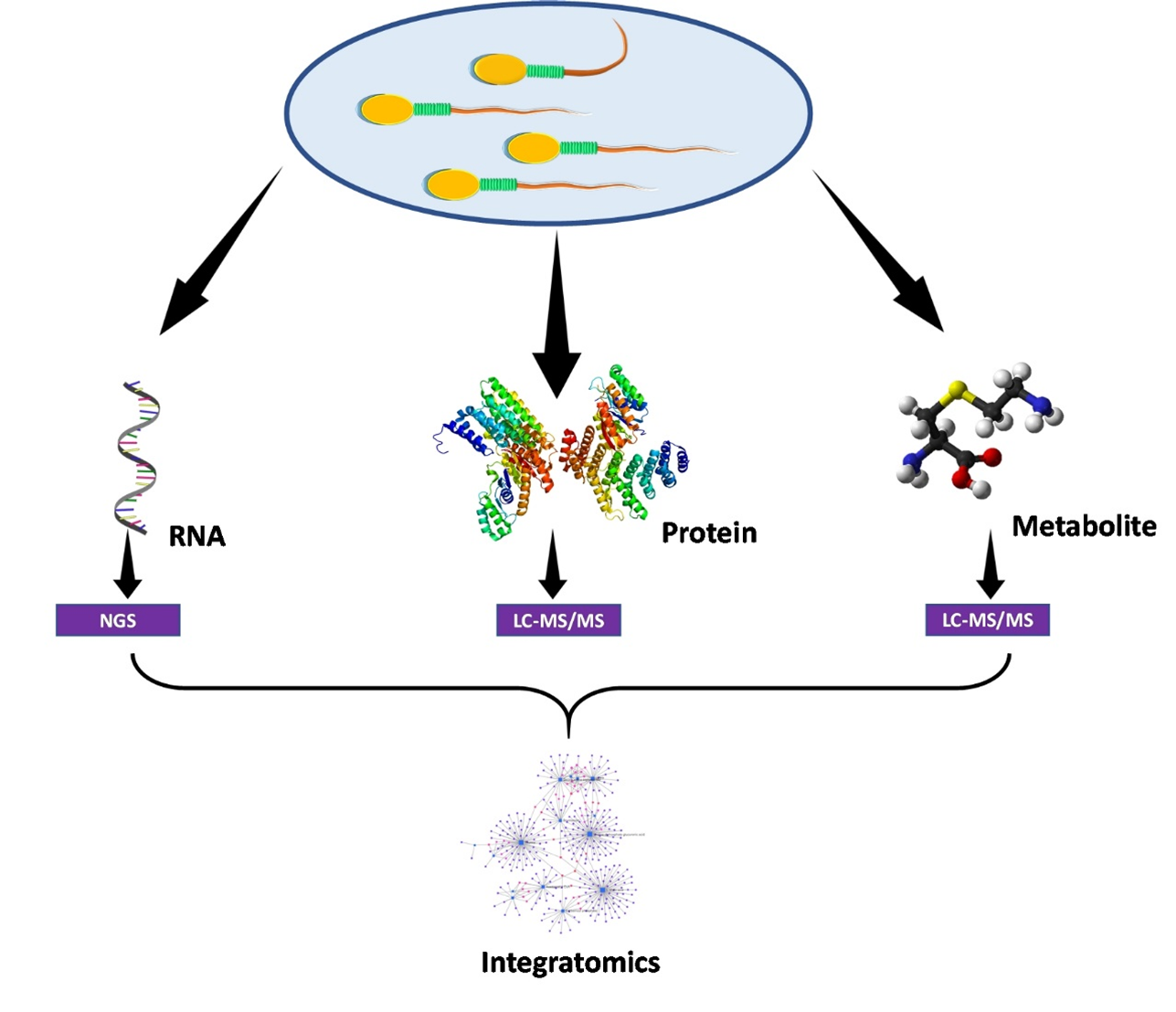


**Fig 6.** Schematic representation of multi-*omic* approach for deciphering the crossbred bull fertility. (NGS: next generation sequence; LC-MS/MS: Liquid Chromatography with tandem mass spectrometry)
